# Oncogenic KRAS, Mucin 4, and Activin A‐Mediated Fibroblast Activation Cooperate for PanIN Initiation

**DOI:** 10.1002/advs.202301240

**Published:** 2023-11-14

**Authors:** Chun‐Mei Hu, Chien‐Chang Huang, Min‐Fen Hsu, Hung‐Jen Chien, Pei‐Jung Wu, Yi‐Ing Chen, Yung‐Ming Jeng, Shiue‐Cheng Tang, Mei‐Hsin Chung, Chia‐Ning Shen, Ming‐Chu Chang, Yu‐Ting Chang, Yu‐Wen Tien, Wen‐Hwa Lee

**Affiliations:** ^1^ Genomics Research Center Academia Sinica Taipei 11529 Taiwan; ^2^ Biomedical Translation Research Center Academia Sinica Taipei 11529 Taiwan; ^3^ Department of Pathology National Taiwan University Hospital Taipei 10041 Taiwan; ^4^ Graduate Institute of Pathology, College of Medicine National Taiwan University Taipei 10041 Taiwan; ^5^ Department of Medical Science National Tsing Hua University Hsinchu 30013 Taiwan; ^6^ Department of Pathology National Taiwan University Hospital−Hsinchu Branch Hsinchu 30331 Taiwan; ^7^ Department of Internal Medicine National Taiwan University Hospital Taipei 10041 Taiwan; ^8^ Department of Surgery National Taiwan University Hospital Taipei 10041 Taiwan; ^9^ Drug Development Center China Medical University Taichung 40402 Taiwan; ^10^ Department of Biological Chemistry University of California Irvine CA 92697 USA

**Keywords:** αSMA^+^ fibroblast, activin A, PanIN, Kras, Muc4

## Abstract

Over 90% of patients with pancreatic ductal adenocarcinoma (PDAC) have oncogenic *KRAS* mutations. Nevertheless, mutated *KRAS* alone is insufficient to initiate pancreatic intraepithelial neoplasia (PanIN), the precursor of PDAC. The identities of the other factors/events required to drive PanIN formation remain elusive. Here, optic‐clear 3D histology is used to analyze entire pancreases of 2‐week‐old *Pdx1*‐*Cre*; *LSL*‐*Kras* ^G12D/+^ (KC) mice to detect the earliest emergence of PanIN and observed that the occurrence is independent of physical location. Instead, it is found that the earliest PanINs overexpress Muc4 and associate with αSMA^+^ fibroblasts in both transgenic mice and human specimens. Mechanistically, *Kras* ^G12D/+^ pancreatic cells upregulate Muc4 through genetic alterations to increase proliferation and fibroblast recruitments via Activin A secretion and consequently enhance cell transformation for PanIN formation. Inhibition of Activin A signaling using Follistatin (FST) diminishes early PanIN‐associated fibroblast recruitment, effectively curtailing PanIN initiation and growth in KC mice. These findings emphasize the vital role of interactions between oncogenic *Kras* ^G12D/+^‐driven genetic alterations and induced microenvironmental changes in PanIN initiation, suggesting potential avenues for early PDAC diagnostic and management approaches.

## Graphical abstract

This study found that early PanIN cells express elevated levels of Muc4, specifically the oncogenic Muc4/X variant, and are closely associated with αSMA+ fibroblasts. This is observed in Kras^G12D/+^ transgenic mice and human pancreatic specimens with early PanINs. Importantly, upregulated Muc4 expression and Activin A secretion are identified as critical factors driving PanIN initiation in pancreatic cells with Kras^G12D/+^ mutation.

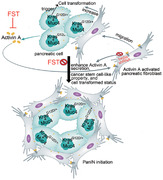

## 1 Introduction

Genetic alterations play a pivotal role in cancer progression. The discovery of oncogenes and tumor suppressor genes provided a framework for elucidating genetic events in cancer evolution. Major advances in genomic sequencing allowed tumor samples from different pathological stages to be sequenced, establishing associations between specific genetic changes and stages of tumor progression. This is particularly the case with pancreatic ductal adenocarcinoma (PDAC). It was noted that two categories of pancreatic intraepithelial neoplasia (PanIN), the precursor of PDAC, including low‐grade (PanIN‐1A, 1B, and 2) and high‐grade (PanIN‐3), were characterized by nuclear and cellular abnormalities prior to becoming full‐blown PDAC.^[^ ^1^ ^]^ The *KRAS* mutation was the earliest and most common genetic alteration observed in more than 90% of low‐grade PanIN. This was followed by inactivation of other tumor suppressor genes, including *CDKN2A*, *TP53*, and *SMAD4*, to trigger PanIN progression to PDAC formation.^[^ ^2^ ^]^

The discovery of KRAS oncogene was a milestone for human cancer genetics.^[^ ^3^ ^]^ The oncogenic activity was demonstrated by transfection of the mutated KRAS into NIH3T3 fibroblasts leading to transformation in vitro and tumor formation in xenografted mice model.^[^ ^4^ ^]^ The presence of the oncogenic *Kras* ^G12D/+^ mutation is crucial for PanIN formation and the development of PDAC.^[^ ^5^ ^]^ However, in Pdx1‐Cre; LSL‐*Kras* ^G12D/+^ (KC) mice at 2.25 months of age, while all pancreatic cells carry the *Kras* ^G12D/+^ mutation, over 80% of the ducts remain normal,^[^ ^6^ ^]^ suggesting that additional factors are required for PanIN formation in the presence of the oncogenic *Kras* ^G12D/+^ mutation. Numerous genetic alterations including mucin overexpression have been associated with PDAC development,^[^ ^2^ ^]^ and several signaling pathways (such as MAPK^[^ ^7^ ^]^ and Wnt/β‐catenin^[^ ^8^ ^]^) and transcription factors (such as Sox9)^[^ ^9^ ^]^ have been reported to be involved in PanIN formation. However, the precise role of these genetic alterations in the earliest PanIN formation remains uncertain.

Mucins are glycoproteins expressed on the apical surfaces of polarized epithelial cells, either as transmembrane or secretory forms, characterized by high molecular weight and extensive O‐linked glycans.^[^ ^10^ ^]^ The presence of polymorphic Variable Number of Tandem Repeats (VNTR) rich in Proline, Threonine and Serine residues is a hallmark of all mucins.^[^ ^11^ ^]^ Splice variants and mutations in mucin genes have been identified in various cancers, including PDAC, and are known to contribute to cancer progression and metastasis.^[^ ^10^, ^12^ ^]^ Notably, MUC4, MUC1, and MUC5AC are expressed as early as the PanIN stage and their expression levels increase further with disease progression.^[^ ^13^ ^]^ Among these mucins, truncated splice variants of MUC4, such as the membrane‐bound MUC4/X^[^ ^14^ ^]^ and MUC4/Y,^[^ ^15^ ^]^ which lack the VNTR (exon 2 domain), have been implicated in promoting tumorigenesis and metastasis. A recent study using a mouse model of spontaneous PDAC lacking Muc4 expression has demonstrated delayed PanIN formation and PDAC development, underscoring the crucial role of Muc4 in PDAC progression.^[^ ^16^ ^]^ However, the involvement of MUC4 expression in oncogenic KRAS‐mediated PanIN initiation remains elusive.

Moreover, besides genetic changes, the microenvironmental changes mediated by oncogenic KRAS also play critical roles in cancer progression.^[^ ^17^ ^]^ Studies on pancreatic injury have demonstrated that environmental alterations can collaborate with mutant Kras to accelerate early neoplasia and neoplastic transformation through epigenetic remodeling, indicating that gene‐environment interactions can influence neoplastic commitment.^[^ ^18^ ^]^ However, the specific genetic and microenvironmental factors that cooperate with oncogenic KRAS to initiate the earliest PanIN formation remain largely unexplored.

In this study, we found that the earliest PanIN cells overexpress *Muc4*, particularly oncogenic *Muc4*/X, and associate with αSMA^+^ fibroblasts in *Kras* ^G12D/+^ transgenic mice as well as in human pancreatic specimens with early PanINs. Upregulation of *Muc4* and Activin A secretion is essential for driving PanIN formation from pancreatic cells with an oncogenic *KRAS* mutation.

## 2 Results

### 2.1 The Earliest PanINs are Randomly Distributed in the *Kras^G12D^* ^/+^ Pancreas

**Figure 1:**
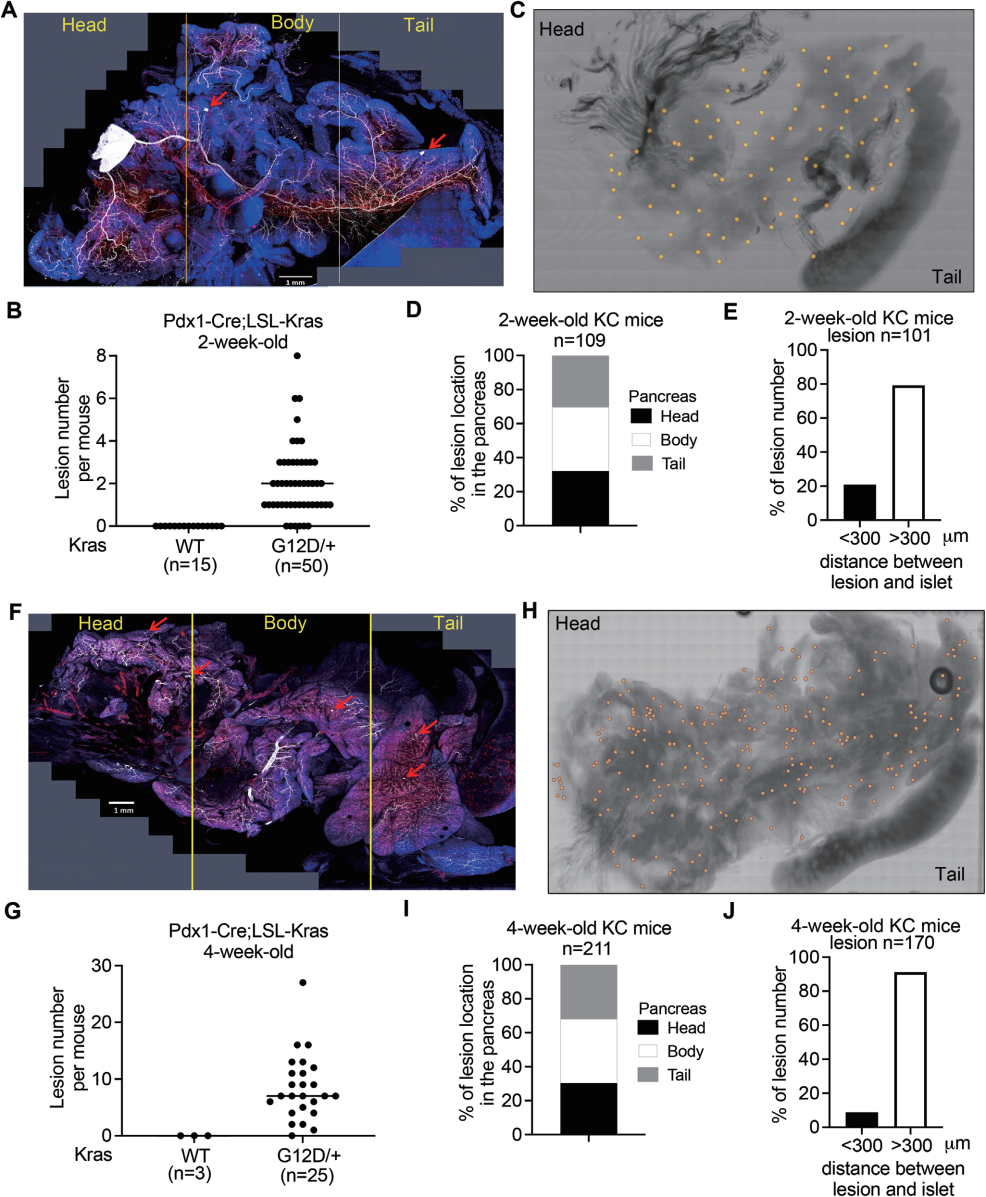
Definition of the earliest PanINs and their physical location in the pancreas of KC mice. A and F) The representative 3D imaging projection of the whole pancreas of 2 (A)‐ and 4 (F)‐week‐old *Pdx1*‐Cre; LSL‐*Kras* ^G12D/+^ (KC) transgenic mouse using 3D histological analysis. Red arrow: the early PanIN. Blue signal: the nucleus staining; White signal: CK19‐staining; Red signal: the blood vessel staining. Scale bar: 1 mm. B and G) Quantification of PanIN number in 2 (B)‐ and 4 (G)‐week‐old transgenic mice. Each dot represents the datum of one mouse. Values were presented as mean ± SD. C and H) The distribution of the earliest PanIN lesions from 45 and 24 of 2 (C)‐ and 4 (H) ‐week‐old KC mice, respectively. One yellow dot indicates one lesion. n = 109 (C); n = 211 (H). D and I) Stacked bar plot showing the percentage of PanIN in the pancreas's head, body, and tail of 2 (D)‐ and 4 (I)‐week‐old KC mice. E and J) Quantification of PanIN and islet association in the pancreas of 2 (E)‐ and 4 (J) ‐week‐old KC mice. An association is defined by the distance between lesion and islet within 300 µm.

Using a whole‐organ visualization method is a valuable strategy for studying pathological lesions.^[^ ^19^ ^]^ Nonetheless, immersing the entire organ in organic solvents such as methyl salicylate carries the risk of organ shrinkage and distortion of the original morphology, particularly in the delicate pancreatic tissue (Figure S1, Supporting Information). To define the earliest PanIN, we performed an optic‐clear 3D histological analysis of the entire pancreas in 2‐week‐old *Pdx1*‐*Cre*; *LSL*‐*Kras* ^+/+^ (control) and *Pdx1*‐*Cre*; *LSL*‐*Kras* ^G12D/+^ (KC) mice using an aqueous solution called RapiClear (Figures S1 and S2A, Supporting Information). In fifty 2‐week‐old KC mice, the number of lesions per mouse numbered 0 to 8, and the average number of lesions per mouse was 2 (**Figure** **1**A,B). Five KC mice had no detectable lesions in their pancreases, as in the 15 control mice, suggesting that the earliest PanIN can be detected in 2‐week‐old KC mice (Figure 1B). We plotted the 109 lesions identified in 45 KC mice onto a representative pancreas image map (Figure 1C) and observed that they exhibited a random distribution and were not associated with endocrine islets, which are known to be involved in complex formation with PanIN during advanced stages of duct lesion development (Figure 1D,E).^[^ ^20^ ^]^ In addition, twenty‐five 4‐week‐old KC mice were also analyzed; the number of lesions per mouse ranged between 0 and 27, and the average number per mouse was 7 (Figure 1F,G). One of the 25 KC mice had no lesions, as did all three control mice (Figure 1G), suggesting that 4‐week‐old KC mice are also suitable for investigating the early process of PanIN formation. Interestingly, the detected lesions in 4‐week‐old KC mice numbered twice more than those observed in 2‐week‐old KC mice (Figure 1B,G), implying that initiation of PanIN may require the accumulation of specific additional mutations over time. Furthermore, we observed similar results indicating a near‐random distribution of early PanINs in 4‐week‐old KC mice as well. Taken together, the results from 2‐ and 4‐week‐old KC mice collectively demonstrate that the earliest PanIN lesions display a random distribution, which contrasts with the preferential occurrence of PDAC in the pancreatic head (58%).^[^ ^21^ ^]^ These data imply that intrinsic genetic alterations may precede microenvironmental influences in the initiation of PanINs.

### 2.2 Muc4 Overexpression in *Kras^G12D^* ^/+^ Pancreatic Cells in the Earliest PanIN

**Figure 2:**
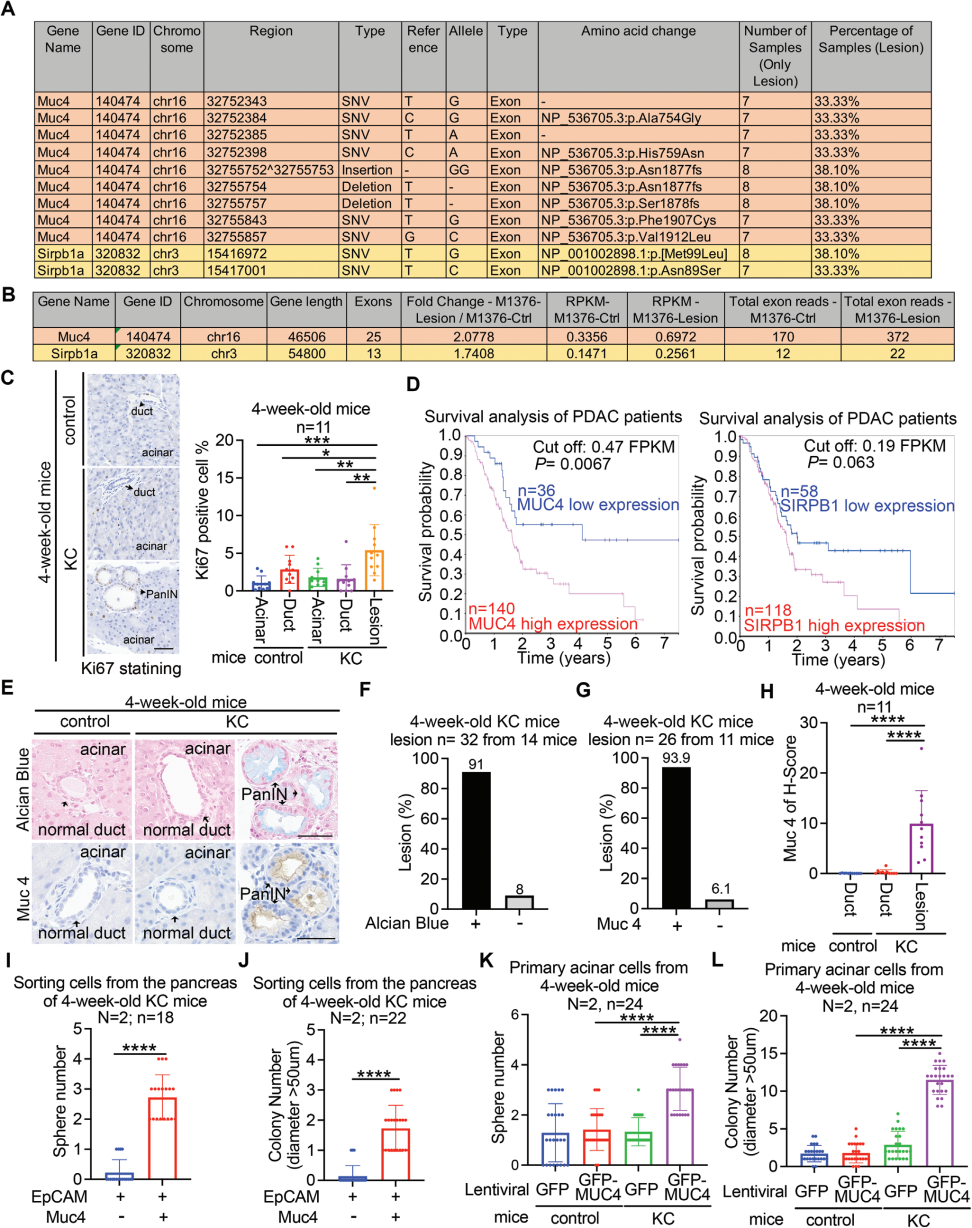
Genetic alterations in the earliest PanIN and *Muc4* up‐regulation cooperate with oncogenic *Kras* ^G12D^ for PanIN initiation. A) The most common genetic alterations in the earliest PanINs from the pancreas of 2‐week‐old KC mice. The data analyzed in this study were obtained from 21 lesions in 13 mice, which were compared to the normal pancreatic region within the same KC mouse. In addition, two control mice were included for comparison. To ensure accuracy, all mouse‐specific single nucleotide polymorphisms (SNPs) were excluded by referencing the UCSC Genome Browser website. B) The RNA‐seq data of the *Muc4* gene and *Sirpb1a*, comparing the PanIN sample with the control sample from a 2‐week‐old KC mouse (M1376). C) The IHC staining of Ki67 proliferation marker. Representative images (left panel) and quantification (right panel) of Ki67 staining in 4‐week‐old control and KC mice. Bar, 50 µm. Each dot represents the datum of one mouse. Values were presented as mean ± SD, n = 11 mice. *, *P*<0.05; **, *P*<0.01; ***, *P* < 0.001 (two‐tailed Student's t‐test). D) Plots of the survival probability of pancreatic cancer patients with *MUC4* (left panel) and *SIRPB1*(right panel) mRNA expression levels from the Human Protein Atlas as shown in methods. *P* values are calculated by log‐rank test. E–H) The Alcian Blue staining and IHC analysis with anti‐Muc4 antibody in 4‐week‐old control and KC mice. Representative images of IHC staining with Alcain Blue and Muc4 E), quantification of Alcain Blue F) and Muc4 G) in the early PanIN cells. Bar, 50 µm. The H score of Muc4 expression in 4‐week‐old control and KC mice H). Each dot represents the datum of one mouse. Values were presented as mean ± SD, n = 11 mice. ****, *P*< 0.0001 (two‐tailed Student's t‐test). I and J) EpCAM^+^/Muc4^−^‐ or double EpCAM/Muc4‐positive pancreatic cells isolated by FACS from 4‐week‐old KC mice for spheroid formation analysis I) and soft agar colony formation analysis J). N indicates independent experiments, and n indicates total measurements in all experiments. Each dot represents the datum from one measurement. Values show mean ± SD. ****, *P*< 0.0001 (two‐tailed Student's t‐test). K and L) Primary acinar cells were isolated from 4‐week‐old control or KC mice for spheroid formation analysis K) and soft agar colony formation analysis L). N indicates independent experiments, and n indicates total measurements. Each dot represents the datum from one measurement. Values show mean ± SD. ****, *P*< 0.0001 (two‐tailed Student's t‐test).

To elucidate the mechanism of how *Kras^G12D/+^* mediated the earliest PanIN formation, we performed manual microdissection based on 3D images (Figure S3A,B, Supporting Information) to capture the earliest PanIN cells and the adjacent phenotypically normal *Kras* ^G12D/+^ acinar cells (control) in the 2‐week‐old KC mice. Systematic genetic analysis by whole‐exome sequencing (WES) (Figure S3C,D, Supporting Information) and gene profiling analysis using RNA‐seq analysis (Figure S4A, Supporting Information) were performed. To investigate the genetic variations in the PanIN cells, we conducted a comparative analysis of WES data between the PanIN region and normal pancreatic region within the same KC mouse, as well as genomic data from two normal pancreatic regions obtained from control mice. To ensure accuracy, we carefully filtered out all mouse‐specific single nucleotide polymorphisms (SNPs) by referring to the UCSC Genome Browser website (https://genome.ucsc.edu/cgi‐bin/hgTrackUi?db = mm10&g = strainSNPs). Our analyses revealed frequent mutations in the *Muc4* and *Sirpb1a* genes in the earliest PanIN cells (**Figure** **2**A). Additionally, RNA sequencing analysis showed upregulation of these two genes in PanIN cells (Figure 2B). Interestingly, we observed the activation of several pathways, such as JNK, ERK1/2, and MAPK (Figure S4B, Supporting Information), which are associated with the oncogenic function of MUC4 in cell survival and proliferation.^[^ ^22^ ^]^ Consistently, PanIN cells exhibited higher levels of Ki67 expression compared to other *Kras* ^G12D/+^ pancreatic ductal cells and *Kras* ^+/+^ pancreatic ductal cells (Figure S2B, Supporting Information; Figure 2C). Importantly, high expression levels of *MUC4*, but not *SIRPB1*, were significantly correlated with poor survival in PDAC patients (Figure 2D). These findings suggest that *MUC4* overexpression may play a crucial role in the initiation of PanIN.

To investigate the potential cooperation between *Kras* ^G12D/+^ and *Muc4* overexpression in triggering PanIN initiation, we initially utilized Alcian blue staining to visualize total mucin content and performed immunohistochemistry (IHC) staining to assess the expression levels of Muc4, Muc5ac, Muc1, and Muc6, all of which were previously observed to be present in the early PanIN (Figure 2E; Figure S5, Supporting Information).^[^ ^13^, ^23^ ^]^ Our observations revealed that over 90% of early PanINs, but not normal pancreatic cells, exhibited favorable Alcian blue staining (Figure 2F) and Muc4 expression (Figure 2G). Additionally, a subset of PanINs displayed Muc5ac, Muc1, and Muc6 expression (Figure S5, Supporting Information). These results imply that Muc4 is the predominant mucin within early PanINs. Additionally, we observed higher expression of Muc4 in PanINs of KC mice compared to pancreatic duct cells of KC and control mice (Figure 2H). Similarly, MUC4 but not MUC5AC was found to be overexpressed in the majority of early PanINs in a human pancreatic specimen, which predominantly consisted of small‐sized PanINs suggestive of early‐stage lesions (Figure S6, Supporting Information), as well as in stage I/II of PDAC tumor specimens (Figure S7, Supporting Information). These findings suggest that MUC4 overexpression may play a role in the initiation of PanINs and their progression to PDAC formation. To affirm the role of Muc4 overexpression in PanIN initiation, we isolated duct‐like cells from *Kras* ^G12D/+^ mice, which included acinar‐to‐ductal metaplasia (ADM) cells and potentially encompassed PanIN cells. Through Western blot analysis, we observed increased expression of Muc4 in these cells compared to Kras^+/+^ ADM cells and Kras^G12D/+^ acinar cells. The predominant band detected in the analysis exhibited a molecular weight of ≈130 kDa (Figure S8A,B, Supporting Information). This 130 kDa Muc4 shares a similar molecular weight with human oncogenic MUC4, MUC4/X and MUC4/Y.^[^ ^12a^ ^]^ Unlike human *MUC4*, mouse *Muc4* is less characterized. Based on human *MUC4* organization, we designed primer sets to analyze the possible expression of *Muc4* variants in 4‐week‐old KC mice (Figure S8C, Supporting Information), and found that duct‐like cells express more *Muc4*, including *Muc4/X* and *Muc4/Y*, than acinar cells (Figure S8D, Supporting Information). These data suggest that Muc4 overexpression, particularly oncogenic Muc4 variants, may have a role in early PanIN formation.

EpCAM is a widely used marker for epithelial cells and tumor cells,^[^ ^24^ ^]^ and MUC4 is commonly known to be expressed in early PanIN cells (Figure 2E,G). Consequently, pancreatic cells that exhibit positivity for both EpCAM and MUC4 might be recognized as PanIN cells. To test whether *Kras* ^G12D/+^ in combination with Muc4 overexpression promotes a PanIN‐like phenotype in culture, we employed EpCAM and Muc4 antibodies to selectively isolate pancreatic cells expressing both EpCAM and MUC4 from 4‐week‐old KC mice. These double‐positive cells exhibited a higher prevalence of the oncogenic *Muc4*/X variant (Figure S9, Supporting Information) and demonstrated enhanced capability in forming spheroid and soft agar colonies compared to EpCAM^+^/Muc4^−^ pancreatic cells obtained from 4‐week‐old KC mice (Figure 2I,J). These data suggest that oncogenic *Kras* ^G12D/+^ mutation may synergize with *Muc4*/X overexpression to initiate PanIN formation. To confirm these results, we ectopically expressed GFP or GFP‐MUC4/X in primary pancreatic acinar cells derived from 4‐week‐old KC mice (Figure S10, Supporting Information) using a lentiviral expression system. Overexpression of GFP‐MUC4/X in *Kras* ^G12D/+^ pancreatic acinar cells significantly increased spheroid formation and soft agar colony formation (Figure 2K,L). To further validate whether the presence of oncogenic *Kras* ^G12D^ and Muc4/X overexpression alone is sufficient to induce PanIN formation, we employed retroviral and lentiviral cDNA expression systems to introduce HA‐KRAS^G12D^ and GFP‐MUC4/X into non‐tumorigenic human pancreatic ductal epithelial (HPDE) cells (Figure S11A, Supporting Information). Our observations revealed that these cells exhibited a preference for forming abnormal cysts lacking a lumen, indicating their transformed phenotype (Figure S11B, Supporting Information).^[^ ^25^ ^]^ Furthermore, these cells increased capacity for spheroid formation and soft agar colony formation, suggesting enhanced proliferation and transformation potential (Figure S11C,D, Supporting Information). Collectively, these results support the notion that the combined presence of oncogenic *Kras* ^G12D/+^ and *Muc4* overexpression, particularly oncogenic *Muc4*/X, can promote cell proliferation and transformation, thereby initiating PanIN formation.

### 2.3 αSMA^+^ Fibroblasts Closely Associate with the Earliest PanIN to Promote PanIN Formation

**Figure 3:**
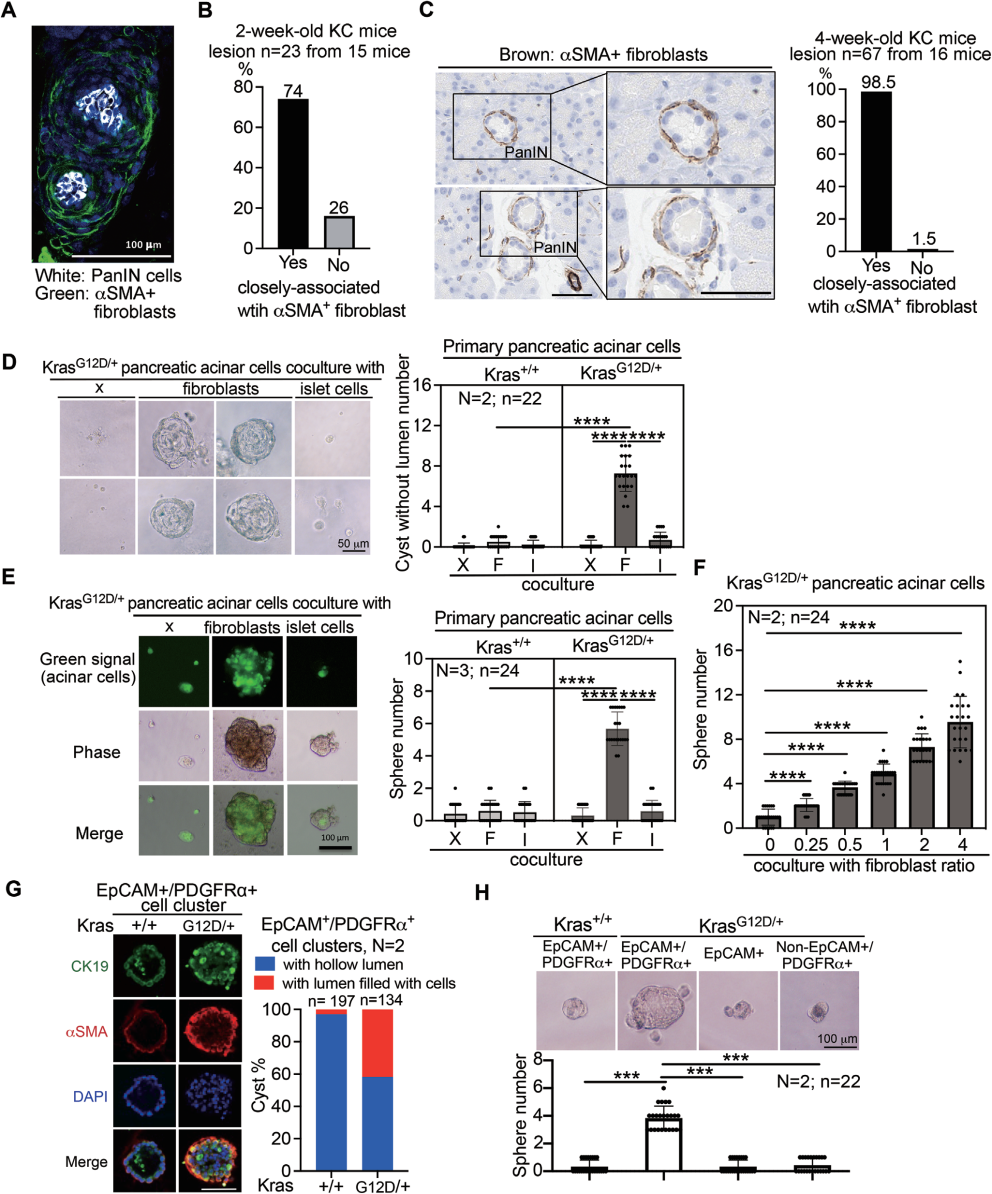
αSMA^+^ fibroblasts associate with the earliest PanIN cells to promote *Kras* ^G12D/+^ pancreatic cell transformation and stemness properties. A) Representative image of 3D histology‐detected the earliest PanINs in the whole pancreas of 2‐week‐old KC mice. Blue signal: the nucleus staining; White signal: CK19‐staining (PanIN cells); Green signal: αSMA staining (activated fibroblasts). Bar, 100 µm. B) Quantification of the percentage of the earliest PanINs associated with αSMA^+^ fibroblasts. C) Representative images of the IHC staining of fibroblasts with anti‐αSMA antibody in 4‐week‐old KC mice (left panel) and quantification of the percentage of early PanINs associated with αSMA^+^ fibroblasts (right panel). Bar, 50 µm. D) Close contact with fibroblasts promotes acinar‐to‐ductal metaplasia of *Kras* ^G12D/+^ pancreatic acinar cells in 3D Matrigel coculture systems. X: *Kras* ^G12D/+^ pancreatic acinar cells only (1000 cells). F: coculture with *Kras* ^+/+^ fibroblasts (2000 cells). I: coculture with *Kras* ^+/+^ islet cells (2000 cells). Upper panel: Representative images in coculture experiment in *Kras* ^G12D/+^ pancreatic cells. Bar, 50 µm. Bottom panel: Quantification of cyst number. E and F) Close contact of *Kras* ^G12D/+^ pancreatic acinar cells with fibroblasts promotes sphere formation. E) 1000 *Kras* ^G12D/+^ pancreatic acinar cells labeled with CellTracker Green CMFDA were cocultured with 2000 Kras^+/+^ fibroblasts or 2000 *Kras* ^+/+^ isle cells for 14 days in a 96‐well low attached plate. (Left panel) Representative images of the cocultured Kras^G12D/+^ pancreatic cells. (Right panel) Quantification of sphere number. Spheres with a diameter ≥ 100 µm were counted. F) 1000 *Kras* ^G12D/+^ pancreatic acinar cells labeled with CellTracker Green CMFDA were cocultured with different ratios of Kras^+/+^ fibroblasts for 14 days in a 96‐well low attached plate. Spheres with a diameter ≥ 100 µm were counted. In the above experiments, N indicates independent experiment, and n indicates total repeated measurements in all experiments. Each dot represents the datum from one measurement. Values were presented as mean ± SD. ****, *P*< 0.0001 (two‐tailed Student's t‐test). G) EpCAM^+^/PDGFRα^+^ cell clusters derived from the pancreas of 4‐week‐old control or KC mice by MACS dissociation and FACS with EpCAM/PDGFRα antibodies for cyst formation analysis. Representative images of cysts without and with lumen were shown in the left panel. CK19 staining indicates pancreatic ductal cells, and αSMA staining indicates activated fibroblasts. Bar, 100 µm. The number of cysts was counted and compared in the right panel. N indicates independent experiment and n indicates cyst number. H) The same cell clusters as above, in addition two more controls including EpCAM^+^ and non‐ EpCAM^+^/PDGFRα^+^ clusters from KC mice, were used for sphere formation analysis. Representative images of spheres were shown in the upper panel and the number of spheres were counted (lower panel). Spheres with a diameter ≥ 100 µm were counted. N indicates independent experiment, and n indicates total repeated measurements in all experiments. Each dot represents the datum from one measurement. Values were presented as mean ± SD. ***, *P* < 0.001 (two‐tailed Student's t‐test).

It was noted that oncogenic *KRAS* modulates the microenvironment to facilitate tumor initiation, progression, and metastasis.^[^ ^17^, ^26^ ^]^ Fibroblastic reactions and inflammation are characteristic features of PDAC.^[^ ^27^ ^]^ Among the stromal cell types, cancer‐associated fibroblasts (CAFs) are the most abundant, constituting up to 80% of the tumor mass in pancreatic tumors.^[^ ^28^ ^]^ In a spontaneous mouse model of PDAC, an increase in αSMA^+^ myofibroblasts was observed, and they were found to be associated with pancreatic epithelial abnormalities ranging from ADM to PanIN and ultimately PDAC.^[^ ^29^ ^]^ However, it remains unclear whether the recruitment of αSMA^+^ myofibroblasts or specific immune cells plays a role in the initiation of PanIN. Using 3D pancreatic histological analysis with two specific antibodies (i.e., CK19 antibody to detect PanIN cells and αSMA antibody to identify activated fibroblasts), we found that 74% of the earliest PanIN cells in 2‐week‐old KC mice were closely associated with αSMA^+^ fibroblasts (**Figure** **3**A,B). Similar results were also obtained in the 4‐week‐old KC mice (Figure S12, Supporting Information). Using conventional 2D IHC staining with an anti‐αSMA antibody, we confirmed that 98.5% of early PanINs in 4‐week‐old KC mice were also associated with αSMA^+^ fibroblasts (Figure 3C). Double staining with anti‐CK19 and anti‐αSMA antibodies further validated the result (Figure S13A, Supporting Information). However, CD45 staining indicated that very few immune cells were detected in the PanINs (Figure S13B, Supporting Information). Since the immune system may not be fully matured in 2‐week‐old or 4‐week‐old mice, we used adult *Elas*‐*CreER*; *LSL*‐*Kras* ^G12D/+^ (EK) transgenic mice as described (Figure S1C, Supporting Information) and focused on early PanIN of a size < 100 µm (Figure S13C–E, Supporting Information). About 86% of the early PanINs demonstrated a similar association with αSMA^+^ fibroblasts (Figure S13F, Supporting Information). Using 2D pancreatic histology, we further confirmed that most early PanINs were associated with αSMA^+^ fibroblasts and not CD45^+^ immune cells (Figure S13G,H, Supporting Information). Furthermore, early PanINs in the human pancreatic specimens were also associated with αSMA^+^ fibroblasts and not with CD45^+^ immune cells (Figure S14, Supporting Information). These results strongly suggest that the activation/recruitment of αSMA^+^ fibroblasts is a major microenvironment change related to the initiation of PanIN.

PDAC may originate from either acinar or ductal cells, and ADM is one of the primary origins of pancreatic pre‐neoplastic lesions that eventually develop into PDAC.^[^ ^30^ ^]^ To explore the biological significance of the above‐observed fibroblast association in PanIN, we employed a 3D Matrigel coculture system in non‐collagen coated plates. This system allowed us to examine whether close contact between *Kras^G12D/+^* pancreatic acinar cells and fibroblasts stimulates the formation of ADM and PanIN. Notably, only ADM and PanIN cells, rather than acinar cells, were capable of forming cysts in this experimental setup. Remarkably, PanIN cells exhibited the unique ability to form cysts with the lumen filled with cells, a feature distinct from normal ducts or cells forming cysts with hollow lumens. This highlights their intrinsic potential for cellular transformation.^[^ ^25^, ^31^ ^]^ Building upon previous findings suggesting the association of the PanIN‐Islet complex with actively dividing epithelial and stromal cells during advanced stages of duct lesion formation,^[^ ^20^ ^]^ we employed endocrine islet cells as a control group and compared their impact on PanIN initiation with that of pancreatic fibroblasts, specifically pancreatic stellate cells (PSCs). As shown in Figure 3D, direct coculture with PSCs, but not islet cells, derived from *Kras* ^+/+^ mice, promoted cyst formation with the lumen filled with cells in *Kras* ^G12D/+^ pancreatic acinar cells, but not *Kras* ^+/+^ pancreatic acinar cells. Furthermore, we used the coculture system to conduct a 3D spheroid assay to measure cancer stem cell‐like properties. In Figure 3E, PSCs, but not islet cells, promoted spheroid formation of *Kras* ^G12D/+^ pancreatic acinar cells. Increasing the number of PSCs enhanced spheroid formation of *Kras* ^G12D/+^ pancreatic acinar cells in a dose‐dependent manner (Figure 3F). In addition, we isolated duct‐like cell‐fibroblast clusters from 4‐week‐old KC mice using mechanical dissociation and Fluorescence‐Activated Cell Sorting (FACS) with anti‐EpCAM/PDGFRα antibodies (Figure S15, Supporting Information) for cyst formation analysis and 3D spheroid assay. Our observations revealed that double EpCAM/PDGFRα‐positive cells exhibited a higher incidence of abnormal cysts with the lumen filled with cells (Figure 3G) and displayed an increased tendency for spheroid formation (Figure 3H) compared to cells from the control mice. These findings strongly indicate that the association with fibroblasts enhances the ability of *Kras* ^G12D/+^ pancreatic cells to form PanIN.

### 2.4 Muc4 Overexpression of *Kras* ^G12D/+^ Pancreatic Cells Promotes Fibroblast Activation/Recruitment in PanIN

**Figure 4:**
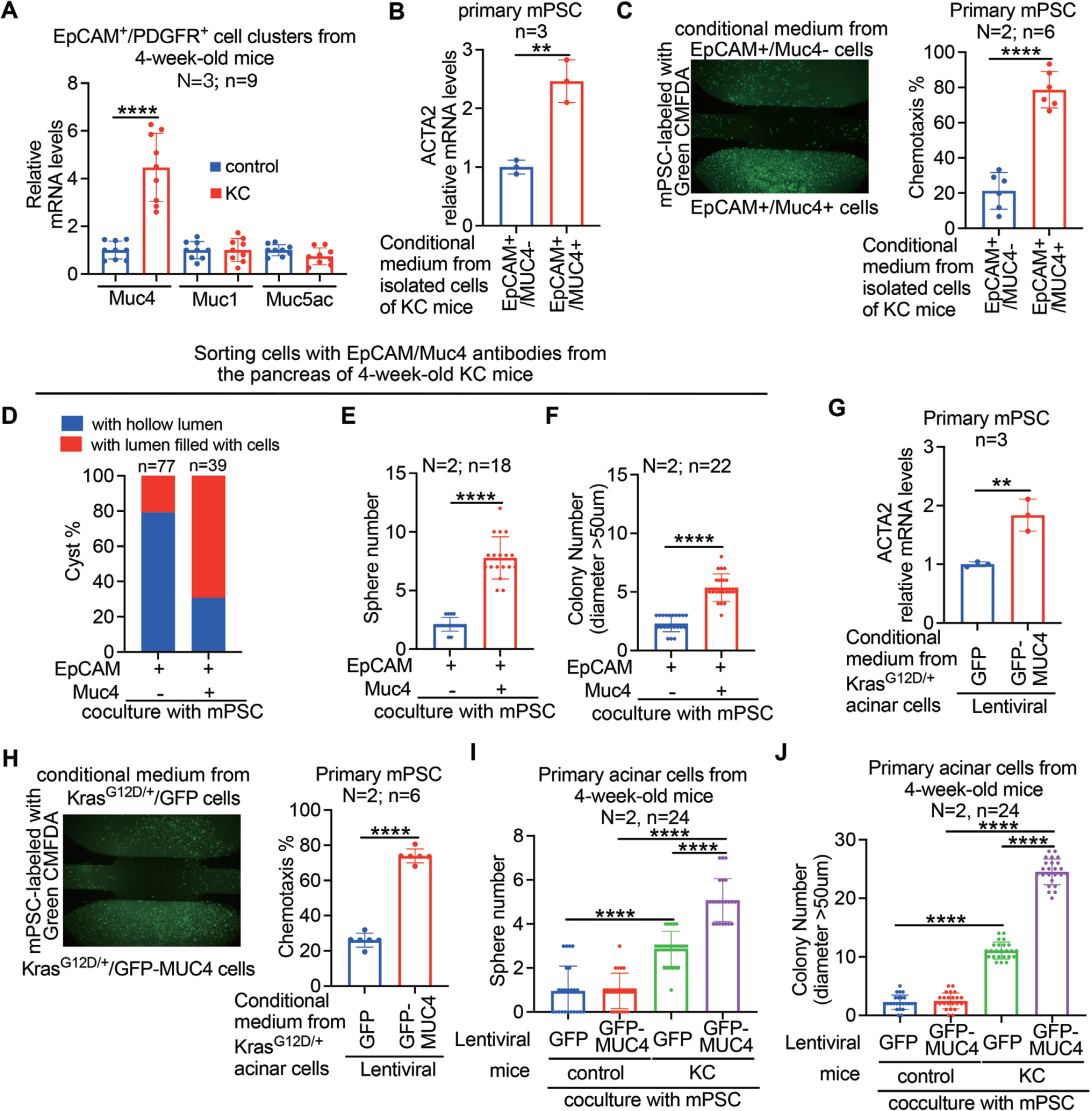
Muc4 overexpression in *Kras* ^G12D/+^ pancreatic cells promotes fibroblast activation and recruitment in the earliest PanIN. A) The mRNA levels of *Muc4, Muc1, and Muc5a* in the EpCAM^+^/PDGFRα^+^ clusters derived from the pancreas of 4‐week‐old KC mice were measured and compared with that of the control mice after normalized with the control gene, *GAPDH*. Primary mPSCs in all experiments were isolated from the pancreas of 4‐week‐old control mice. For chemotaxis analysis, mPSCs were labeled with CellTracker Green CMFDA for 10 mins before the experiment. B) Expression of *ACTA2* gene in mPSC treated with conditional media derived from EpCAM^+^/Muc4^−^ or EpCAM^+^/Muc4^+^ pancreatic cells isolated by FACS from 4‐week‐old control or KC mice by qPCR. C) Left panel: Representative images of µslide chemotaxis analysis of mPSC after treated with conditional media as B). Right panel: Quantitation of the percentage of chemotaxis. D–F) EpCAM^+^/Muc4^−^‐ or EpCAM^+^/Muc4^+^‐pancreatic cells were cocultured with mPSC cells for cyst formation analysis D), sphere formation analysis E), and soft colony formation analysis F). For sphere formation analysis, sphere with a diameter ≥ 100 µm were counted. G) Expression of *ACTA2* gene in mPSC treated with conditional media derived from pancreatic acinar cells isolated from 4‐week‐old control or KC mice by qPCR. These primary pancreatic acinar cells were infected with lentiviral GFP or lentiviral GFP‐MUC4 (MUC4/X). H) Left panel: Representative images of µslide chemotaxis analysis of mPSC after treated with conditional media as G). Right panel: Quantitation of the percentage of chemotaxis. I and J) Pancreatic acinar cells from KC or control mice were ectopically expressed GFP‐MUC4/X or GFP only and cocultured with mPSC cells for sphere formation analysis I) and soft colony formation analysis J). For soft agar colony formation analysis, the colony with a diameter ≥ 50 µm was counted. N indicates independent experiment, and n indicates total repeated measurements in all experiments. Each dot represents the datum from one measurement. Values were presented as mean ± SD. **, *P* < 0.01; ****, *P*< 0.0001 (two‐tailed Student's t‐test).

To explore the potential interplay between Muc4 overexpression and fibroblast association in PanIN, we isolated double EpCAM/PDGFRα‐positive cell clusters from the pancreases of 4‐week‐old KC mice and control mice. In KC mice, these clusters comprised both PanIN cells and normal duct cells, while in control mice, they exclusively consisted of normal duct cells. Subsequently, we perform qPCR analysis on these cell clusters to assess the expression of mucin genes (*Muc1*, *Muc4*, *Muc5ac*), known to be expressed in low‐grade PanIN stages.^[^ ^13^ ^]^ We found that the double EpCAM/PDGFRα‐positive cell clusters of KC mice exhibited higher mRNA expression of *Muc4*, but not *Muc1* and *Muc5ac*, compared to those from control mice (**Figure** **4**A). This discovery is consistent with a study conducted by Mallya et al., which indicated that Muc4 expression precedes the expression of Muc1 and Muc5ac in the early PanIN of the iKC mouse model.^[^ ^13b^ ^]^ Importantly, these results indicated that *Muc4* is the first mucin to be expressed and up‐regulated in early PanIN associated with fibroblasts. We hypothesized that cooperation between *Kras* ^G12D/+^ and Muc4 overexpression, particular oncogenic *Muc4*/X, in pancreatic cells could activate and recruit PSCs for PanIN formation. To test this hypothesis, we initially isolated two types of cells from the pancreases of 4‐week‐old KC mice: double‐positive cells for EpCAM and Muc4, which exhibited high expression of *Muc4*/X variant, and EpCAM‐positive/Muc4‐negative cells. We then collected conditioned media from these cells and used it to treat primary PSCs derived from 4‐week‐old control mice. To examine fibroblast activation by measuring *αSMA* gene (*ACTA2*) expression, we observed that the conditioned media derived from double EpCAM/Muc4‐positive pancreatic cells exhibited a greater capacity to induce *ACTA2* gene expression (Figure 4B). Moreover, to investigate the effects of fibroblast recruitment, we conducted PSC chemotaxis assays using conditioned media, and observed that conditioned media derived from double EpCAM/Muc4‐positive pancreatic cells exhibited a greater tendency to attract PSCs compared to conditioned media from EpCAM‐positive/Muc4‐negative pancreatic cells (Figure 4C). In addition, to explore the interplay among *Kras* ^G12D/+^, *Muc4* overexpression, and fibroblast association in promoting PanIN formation, we conducted coculture experiments. As depicted in Figure 4D–F, our results revealed that fibroblast association significantly enhanced the capacity of double EpCAM/Muc4‐positive pancreatic cells, but not EpCAM‐positive/Muc4‐negative pancreatic cells, to form cysts without lumens, spheroids, as well as colonies in soft agar. To validate these findings, we employed *Kras* ^G12D/+^ pancreatic acinar cells with ectopic expression of either GFP or GFP‐MUC4/X to collect conditioned media for PSC activation and recruitment analysis. We also conducted coculture experiments using PSCs to assess spheroid formation and soft agar colony formation. Consistent with the results obtained from double EpCAM/Muc4‐positive pancreatic cells, we observed that conditioned media derived from *Kras* ^G12D/+^ pancreatic acinar cells ectopically expressing GFP‐MUC4/X exhibited significant effects in increasing *ACTA2* gene expression (Figure 4G) and PSC recruitment (Figure 4H), compared to conditioned media derived from Kras^G12D/+^ pancreatic acinar cells ectopically expressing GFP. Furthermore, coculture of *Kras* ^G12D/+^ pancreatic acinar cells ectopically expressing GFP‐MUC4/X with PSCs resulted in a greater formation of spheroids (Figure 4I) and soft agar colonies (Figure 4J), as compared to coculture of *Kras* ^G12D/+^ pancreatic acinar cells ectopically expressing GFP with PSCs. Overall, these findings collectively demonstrated that the cooperative effect of *Kras* ^G12D/+^ and *Muc4* overexpression, particular oncogenic *Muc4*/X, in pancreatic cells has the potential to activate and recruit fibroblasts for PanIN formation.

### 2.5 Activin A Secreted from Muc4‐Overexpressing/*Kras* ^G12D/+^ Pancreatic Cells Promotes Fibroblast Activation/Recruitment in PanIN

**Figure 5:**
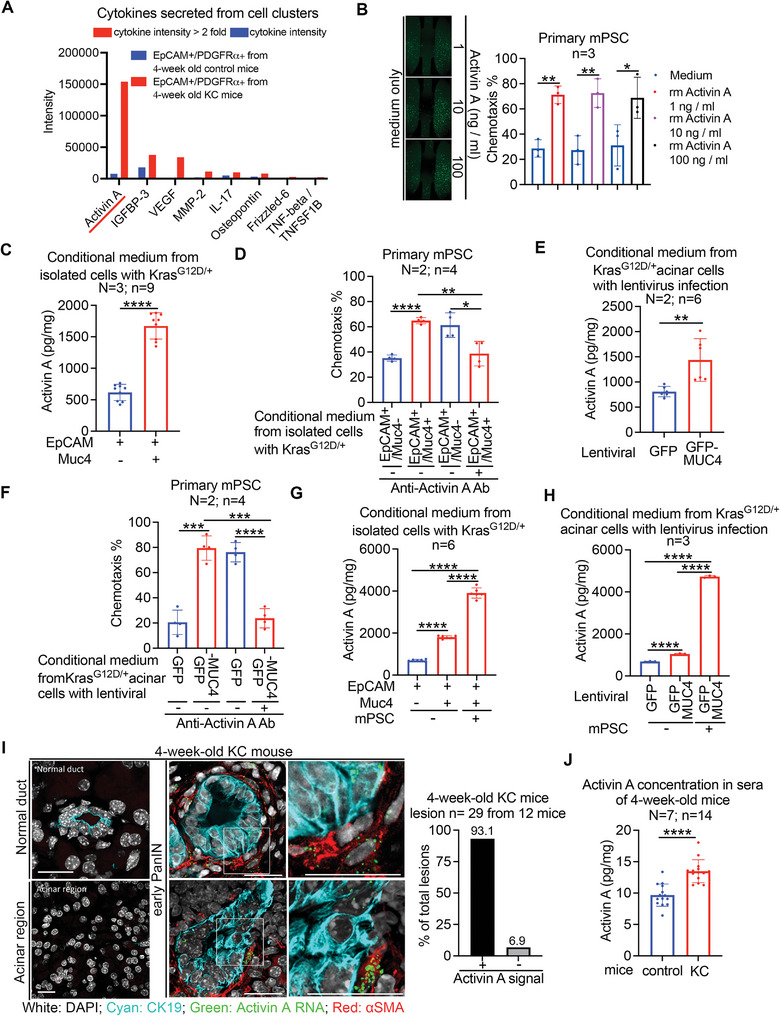
Activin A from Muc4 overexpressed and *Kras* ^G12D/+^ pancreatic cells facilitate fibroblast recruitment for PanIN formation. A) Cytokines analysis of conditional media from the cell clusters isolated from 4‐week‐old control or KC mice using RayBio® Mouse Biotin‐Label Based Antibody Array (Mouse L‐308 Array, Glass Slide). B) The effect of Activin A on mPSC chemotaxis was analyzed using μ‐Slide chemotaxis analysis. Representative images (left panel) and quantification (right panel) of the chemotaxis effect. C) Quantification of Activin A in the conditional media of EpCAM^+^/Muc4^−^‐ or EpCAM^+^/Muc4^+^‐pancreatic cells using ELISA analysis. D) Anti‐Activin A antibody abolishes mPSC chemotaxis. Conditional media from EpCAM^+^/Muc4^+^‐pancreatic cells were pre‐treated with or without 4 µg mL^−1^ of anti‐Activin A antibody for 30 mins and were subjected to µslide chemotaxis analysis. E) Quantification of Activin A in the conditional media of pancreatic acinar cells infected with lentiviral GFP or lentiviral GFP‐MUC4 (MUC4/X) using ELISA. F) Anti‐Activin A antibody abolishes mPSC chemotaxis. Conditional media from lentiviral GFP‐MUC4/X‐infected pancreatic acinar cells were pre‐treated with or without 4 µg mL^−1^ of anti‐Activin A antibody for 30 min and were subjected to μ‐Slide chemotaxis analysis. G) Increases of Activin A secretion of media from FACS‐isolated pancreatic cells cocultured with or without fibroblasts by ELISA analysis. H) Activin A secretion from *Kras* ^G12D/+^ pancreatic acinar cells infected with the indicated lentivirus after cocultured with or without fibroblasts by ELISA analysis. In the above experiments, N indicates independent experiments, and n indicates total repeated measurements in all experiments. Each dot represents the datum from one measurement. Values were presented as mean ± SD. *, *P*< 0.05; **, *P* < 0.01; ***, *P*< 0.001; ****, *P*< 0.0001 (two‐tailed Student's t‐test). I) *Activin A* mRNA expression in PanIN and PanIN‐associated fibroblasts in 4‐week‐old KC mice was detected by an Opal Multiplex IHC Assay. Representative IHC images (left panel) and quantification of Activin A expressed in early PanIN cells (right panel). Bar, 20 µm. White signal: DAPI; Cyan signal: Normal duct cells and PanIN cells stained with anti‐CK19 antibody; Red signal: αSMA^+^ fibroblasts stained with anti‐αSMA antibody; Green dot signal: *Activin A* mRNA with dig‐labeled antisense mRNA probes. Values were presented as mean ± SD, PanIN lesion n = 29 from 12 KC mice. J) Sera from 4‐week‐old control or KC mice were used to perform Activin A ELISA analysis. N = 7 mice; two duplicate experiments for each mouse, n = total 14 repeated measurements. Each dot represents the datum from one measurement. Values were presented as mean ± SD. *, *P*< 0.05 (two‐tailed Student's t‐test).

The results above led us to hypothesize that secreted factors may mediate fibroblast activation/recruitment driving PanIN formation. To address this, we isolated double EpCAM/PDGFRα‐positive cell clusters from the pancreases of 4‐week‐old KC and control mice and collected the respective conditional media for cytokine array analysis. Using the RayBio Biotin label‐based Mouse Antibody Array, we found that Activin A was the highest expressed cytokine in double EpCAM/PDGFRα‐positive cell clusters of KC mice in comparison to control mice (**Figure** **5**A). It was previously reported that Activin A could trigger the activation/migration of skin fibroblasts^[^ ^32^ ^]^ and activate PSCs to myofibroblasts.^[^ ^33^ ^]^ We then examined whether Activin A has chemo‐attractant ability for PSC recruitment using μ‐Slide chemotaxis analysis. This assay revealed that as little as 1 ng mL^−1^ of recombinant Activin A is sufficient to attract both mouse(m) PSCs (Figure 5B) and human(h) PSCs (Figure S16A, Supporting Information). Subsequently, to explore the potential of the cooperative effect between Kras^G12D/+^ and Muc4 overexpression, specifically the oncogenic Muc4/X, in inducing Activin A secretion in pancreatic cells, we isolated double EpCAM/Muc4‐positive pancreatic cells and EpCAM‐positive/Muc4‐negative pancreatic cells from 4‐week‐old KC mice. Using ELISA analysis, we measured the levels of Activin A secretion and observed that double EpCAM/Muc4‐positive pancreatic cells exhibited higher Activin A secretion compared to EpCAM‐positive/Muc4‐negative pancreatic cells (Figure 5C). To investigate whether the recruitment of PSCs by double EpCAM/Muc4‐positive pancreatic cells was mediated through Activin A secretion, we employed neutralizing anti‐Activin A antibodies to treat the conditioned media and performed PSC chemotaxis assays. In Figure 5D, we observed that the ability of conditional media derived from double EpCAM/Muc4‐positive pancreatic cells to recruit mPSCs was significantly diminished upon pre‐treatment with neutralizing anti‐Activin A antibodies. To validate whether the cooperation between *Kras* ^G12D/+^ and *Muc4*/X overexpression triggered PSC recruitment via Activin A, we conducted similar experiments using *Kras* ^G12D/+^ pancreatic acinar cells with ectopic expression of either GFP or GFP‐MUC4/X (Figure 5E,F), as well as HPDE cells expressing HA‐KRAS^G12D^/GFP or HA‐KRAS^G12D^/GFP‐MUC4/X (Figure S16B,C, Supporting Information). Remarkably, the results obtained from these two cell systems were consistent with those observed in double EpCAM/Muc4‐positive pancreatic cells. Collectively, these findings strongly suggest that Muc4/X overexpression induces *Kras* ^G12D/+^ cells to secrete Activin A, thereby activating and recruiting PSCs.

**Figure 6:**
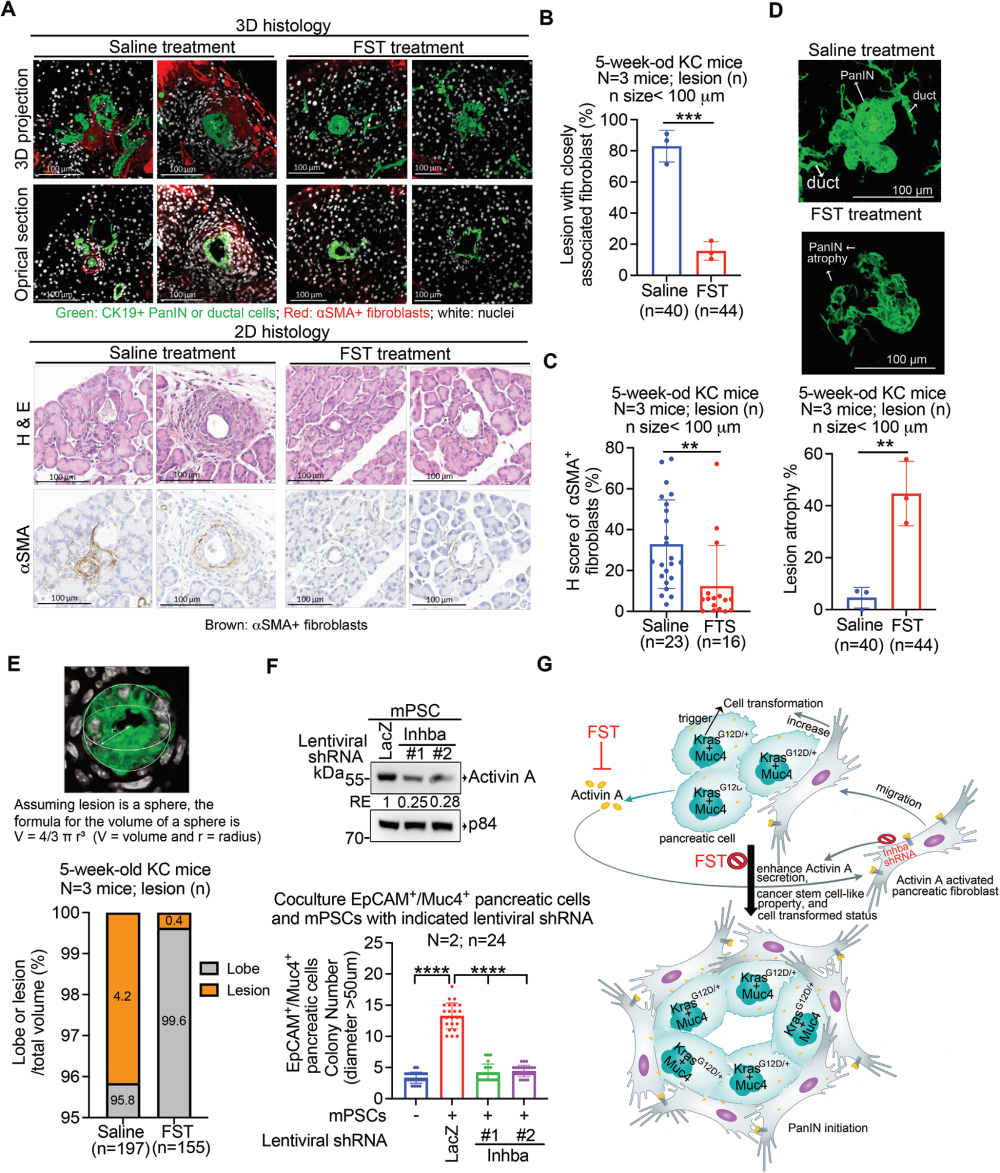
The proposed model for the cooperation of oncogenic *Kras* ^G12D/+^, Muc4 overexpression, and fibroblast activation for PanIN initiation. A–E) 3‐week‐old KC mice were administered either Saline solution or a dose of 1 µg k^−1^ g follistatin (FST) for a duration of two weeks. After the two‐week treatment period, we performed integrated 3D/2D pancreas histology to assess fibroblast recruitment and PanIN formation. A) Representative images depicting 3D and 2D histology of the pancreas. In the 3D staining images, green color represents CK19 staining, indicating PanIN or ductal cells; red color represents αSMA staining, indicating activated fibroblasts; and white color represents DAPI staining, indicating nuclei. In the 2D IHC images, brown color represents αSMA staining, indicating activated fibroblasts. Scale bar: 100 µm. B) The percentage of lesions associated with fibroblast. Each dot represents the datum of one mouse. Values were presented as mean ± SD. Mouse number and lesion number are denoted by N and n, respectively. ***, *P*< 0.001 (two‐tailed Student's t‐test). C) The H score of αSMA^+^ fibroblasts associated with lesion of specified mice. Values were presented as mean ± SD. Mouse number and lesion number are denoted by N and n, respectively. **, *P*< 0.01 (two‐tailed Student's t‐test). D) Representative lesion images (upper panel) and atrophy lesion quantification (lower panel) at KC mice treated with Saline solution or FST. Green color represents CK19 staining, indicating PanIN. Each dot represents the datum of one mouse. Values were presented as mean ± SD. Mouse number and lesion number are denoted by N and n, respectively. **, *P*< 0.01 (two‐tailed Student's t‐test). E) The percentage of lobe and lesions volume in KC mice treated with Saline solution or FST. Mouse number and lesion number are denoted by N and n, respectively. The total tissue volume calculated in the Saline solution group is 279.4 mm^3^, while in the FST group, it is 252.8 mm^3^. F) mPSCs were obtained from 4‐week‐old control mice and subjected to overnight infection with 10 MOI of the specified lentiviral shRNA. After one day of recovery from the virus infection, we selectively enriched lentiviral shRNA‐positive cells by applying puromycin selection at a concentration of 1 µg mL^−1^ for three days. Following a two‐day recovery period, the cells were subjected to Western blot analysis (upper panel) and cocultured with double EpCAM and Muc4‐positive pancreatic cells isolated from 4‐week‐old KC mice for the assessment of soft agar colony formation (lower panel). The number of colonies larger than 50 µm was quantified after a 14‐day coculture period. N indicates independent experiment, and n indicates total repeated measurements in all experiments. Each dot represents the datum from one measurement. Values were presented as mean ± SD. ****, *P*< 0.0001 (two‐tailed Student's t‐test). G) Proposed model of oncogenic Kras^G12D^‐mediated PanIN initiation. The overexpression of Muc4 (Muc4/X) in *Kras* ^G12D/+^ pancreatic cells enhances cell transformation and stimulates Activin A secretion, which in turn activates and recruits' fibroblasts. The activated fibroblasts further promotes Activin A secretion, elevates pancreatic cell transforming status, and enhances cancer stemness properties, thereby contributing to PanIN initiation. The inhibition of Activin A signaling using Follistatin (FST), an Activin A antagonist, effectively suppresses fibroblast recruitment and inhibits PanIN formation. Additionally, reducing Activin A expression in mPSCs through the use of lentiviral Inhba^shRNA^ also impedes PanIN formation.

It was noted that direct contact of pancreatic tumor cells with PSCs promotes Activin A secretion from PSCs to further enhance stemness properties of tumor cells.^[^ ^33^ ^]^ To assess the influence of the interaction between *Kras* ^G12D/+^ and *Muc4*/X‐overexpressing pancreatic cells and PSCs on Activin A secretion, we cocultured PSCs with either *Kras* ^G12D/+^ pancreatic cells or *Kras* ^G12D/+^/*Muc4*/X‐overexpressing pancreatic cells and observed that the combination of PSCs with *Kras* ^G12D/+^/*Muc4*/X‐overexpressing pancreatic cells resulted in enhanced Activin A secretion (Figure 5G,H; Figure S16D, Supporting Information). Additionally, utilizing an Opal Multiplex IHC Assay, we detected Activin A in early PanIN cells and neighboring fibroblasts in 4‐week‐old KC mice (Figure 5I) as well as in a human pancreatic specimen with early PanINs (Figure S17, Supporting Information). Intriguingly, the sera of 4‐week‐old KC mice contained higher levels of Activin A compared to control mice (Figure 5J). To investigate the involvement of Activin A in PanIN formation, we administered intraperitoneal injections of either saline solution or 1 µg k^−1^g follistatin (FST) to 3‐week‐old KC mice for two weeks. Follistatin is known to bind to Activin A with high affinity,^[^ ^34^ ^]^ thereby blocking it signaling. Following the two‐week treatment, we conducted 3D/2D integrative whole pancreas histology and observed that blocking Activin A signaling with FST reduced fibroblast recruitment in early PanIN, resulting in suppressed PanIN formation and growth (**Figure** **6**A–E). Furthermore, to validate the role of recruited fibroblasts in providing Activin A for PanIN formation, we employed lentiviral shRNA to delete Activin A expression from mPSCs and assessed its impact on PanIN formation (Figure 6F). Through soft agar colony formation experiments in the cocultures, we observed that PSCs with decreased Activin A expression exhibited lower capability in promoting the formation of soft agar colonies by double EpCAM/Muc4‐positive pancreatic cells compared to PSCs with *LacZ* ^shRNA^. Collectively, these findings suggest that *Kras* ^G12D/+^ pancreatic cells with *Muc4*/X overexpression secrete Activin A, which activates and recruits PSCs. In turn, the activated PSCs contribute to an increased secretion of Activin A, thereby driving PanIN formation.

## 3 Discussion

In this communication, we employed an aqueous RapiClear solution as a clearing agent to facilitate 3D whole pancreas histology. We compared this new method with the organic solvent (methyl salicylate)‐based FLASH method^[^ ^19^ ^]^ for whole‐organ visualization and found that the newer approach demonstrated superior effects in minimizing organ shrinkage, preserving the original pancreas morphology, and reducing background signals compared to the FLASH method (Figure S1, Supporting Information). Moreover, the tissue could be easily reverted from the 3D immersed state using RapiClear, enabling subsequent 2D H&E staining. This technique proved particularly valuable for investigating the physical location and microenvironment of pathological cell occurrences. Furthermore, through the application of this RapiClear‐based approach to study PanIN initiation, we demonstrated that *Kras* ^G12D/+^ pancreatic cells, through genetic alterations, upregulate *Muc4* expression, particularly oncogenic Muc4/X overexpression, to enhance proliferation. In addition, Activin A secretion is stimulated to activate and recruit fibroblasts, which leads to additional Activin A secretion from these fibroblasts to elevate the transformed status of cells giving rise to PanIN (Figure 6G). Since PanIN 1 is the earliest step of PDAC development, cooperation of *Kras* ^G12D/+^ pancreatic cells with upregulated Muc4/X expression and Activin A secretion, in concert with activated/recruited fibroblasts, appears to be the most critical step for PanIN formation and PDAC initiation.

To explore the potential applications of these findings in the early detection or treatment of PDAC, our study specifically focused on stage I/II PDAC cases, which represent the resectable and early stages of the disease, as well as high‐risk controls (HRCs) who have a family history of PDAC but remained free from pancreatic malignancies for more than 2 years. We observed that PDAC patients had higher serum levels of Activin A compared to HRCs, and these levels correlated with the disease stage (Figure S18A,B, Supporting Information). Additionally, elevated serum Activin A levels were found to be positively associated with poor prognosis in PDAC patients (Figure S18C, Supporting Information). These findings were consistent with the survival analysis of PDAC patients from the Human Protein Atlas database, where mRNA levels of the Activin A gene (*INHBA*) in PDAC tumors were examined (Figure S18D, Supporting Information). Both Figure S18D (Supporting Information) and Figure 2D in our study were derived from the Human Protein Atlas database, which utilized the same PDAC cohort and included a population comprising over 94% of stage I/II PDAC cases. Furthermore, in our analysis of the impact of MUC4 and INHBA mRNA expressions on survival, we observed that PDAC patients with high expression levels of both MUC4 and INHBA had a poorer prognosis compared to the other group (Figure S18E, Supporting Information). Notably, targeting MUC4 or INHBA has shown effectiveness in suppressing PDAC malignancy.^[^ ^33^, ^35^ ^]^ Furthermore, a recent study demonstrated that depleting Muc4 delayed PanIN initiation and PDAC formation in KPC mice.^[^ ^36^ ^]^ In our study, we demonstrated that blocking Activin A signaling using FST could reduce fibroblast recruitment in early PanIN, leading to suppressed PanIN formation and growth in KC mice (Figure 6A to E). Collectively, these findings strongly support the critical role of MUC4 or Activin A in the process of PanIN initiation to PDAC formation. Targeting MUC4 or Activin A presents potential opportunities for early diagnostic and management strategies for PDAC.

MUC4 expression is regulated at both the transcriptional and post‐transcriptional levels. In PDAC, multiple regulators, including oncogenes (i.e., mutant *KRAS*), epigenetic modifications, and tumor microenvironment factors (e.g., TGFβ, IL‐4, IL‐9, IL‐6, interferon‐ɣ, retinoic acid, bile acids, and hypoxia), are reported to induce MUC4 expression.^[^ ^35a^ ^]^ Oncogenic *KRAS* upregulates *MUC4* expression at the transcriptional level through the AP‐1 and NF‐κB transcription factors via MAPK, JNK, and NF‐κB signaling pathways and at the post‐transcriptional level by a mechanism involving the RalB GTPase.^[^ ^37^ ^]^ Interestingly, in the pancreas‐expressed oncogenic *Kras* ^G12D/+^ in transgenic mice, Muc4 expression was detected only in PanIN cells, but not in normal ductal and acinar cells (Figure 2E), suggesting that *Kras* ^G12D/+^ may not directly activate Muc4 expression. Intriguingly, *MUC4* is also upregulated in the *KRAS* ^WT^ PDAC cell line BxPC3, and silencing of *MUC4* affected its malignancy,^[^ ^38^ ^]^ suggesting that oncogenic *KRAS* is not the sole factor affecting *MUC4* overexpression.

*MUC4* contains 26 exons with a very complex gene organization^[^ ^10a^ ^]^ that encodes proteins with molecular weights ranging from 550 to 930 kDa due to the polymorphic Variable Number of Tandem Repeats (VNTR) region in exon 2. MUC4 protein comprises two functional subunits (MUC4α and MUC4β), cleaved at the Gly‐Asp‐Pro‐His (GDPH) site and containing four functionally unique domains termed NIDO, AMOP, vWD, and three EGF‐like domains downstream of the central VNTR region.^[^ ^12a^ ^]^ Twenty‐four distinct splice transcripts of *MUC4* have been isolated from various tissue samples as well as cell lines.^[^ ^39^ ^]^ Four different transcript variants of human *MUC4* (i.e., variant 1, variant 4 (*MUC4/Y*), variant 5 (*MUC4/X*), and variant 6) correspond to four isoforms (a, d, e, and f) found in the NCBI nucleotide database. *MUC4*/Y and *MUC4*/X are two shorter variants of MUC4 that lack exon 2 (VNTR region) and lack exon 2 and exon 3, respectively. Exon 3 does not contain a functional domain. The VNTR region in exon 2 of MUC4 undergoes extensive glycosylation with O‐linked glycans. The highly glycosylated TR domain of MUC4 is believed to impede tumor cell interaction with extracellular matrix (ECM) proteins by obstructing integrin accessibility to ECM ligands in a steric manner.^[^ ^40^ ^]^ Consequently, both *MUC4*/X^[^ ^14^ ^]^ and *MUC4*/Y,^[^ ^15^ ^]^ which lack exon 2, have been implicated in promoting tumorigenesis and metastasis and are recognized to exhibit oncogenic activity. Consistently, we found that Muc4 is overexpressed in ≈90% of Kras^G12D/+^ PanIN cells (Figure 2G). The majority of Muc4 mutations detected in PanIN cells were single nucleotide variations (SNVs) that exclusively occurred in exon 2 of the *Muc4* gene, similar with a previous report identified exon 2 as a frequently mutated exon in the *MUC4* gene.^[^ ^12a^ ^]^ Furthermore, the overexpressed Muc4 protein in *Kras* ^G12D/+^ duct‐like cells, mostly comprising PanIN cells, had a molecular weight of ≈130 kDa (Figure S8A,B, Supporting Information), similar to the size of oncogenic MUC4/X and MUC/Y. Using primers designed to detect *Muc4* variants in qPCR experiments, we further confirmed the upregulation of either *Muc4*/X or *Muc4*/Y in *Kras* ^G12D/+^ duct‐like cells (Figure S8C,D, Supporting Information). Furthermore, we tested the effects of oncogenic *MUC4*/X in PanIN initiation by overexpressing GFP‐MUC4/X in *Kras* ^G12D/+^ pancreatic acinar cells and HA‐KRAS^G12D^/GFP‐MUC4/X in HPDE cells (Figure 2K and L; Figure S11, Supporting Information), and confirmed that the cooperation between oncogenic KRAS^G12D^ and oncogenic MUC4 overexpression, such as MUC4/X, is sufficient to promote cell transformation. Genetic alterations in splicing process in *Kras* ^G12D/+^ cells may contribute to the observed transcriptional heterogeneity in the expression of cancer‐associated *MUC*4.^[^ ^10^, ^12^ ^]^ Although the precise mechanism remains to be explored, the mutations in *Muc*4 may trigger alternative splicing of *Muc*4, resulting in an increase in *Muc4*/X and *Muc4*/Y for PanIN initiation. Thus, targeting MUC4/X and MUC4/Y using specific recombinant β subunit of human MUC4 (rMUC4β)^[^ ^41^ ^]^ would be a worthwhile endeavor for treating PDAC.

In addition to the oncogenic *Kras^G12D/+^*‐induced intrinsic effect resulting in *Muc4* overexpression, a prominent phenotype in all early PanIN cells is the close association with αSMA^+^ fibroblasts, but not immune cells (Figure 3; Figures S13 and S14, Supporting Information). *Kras* ^G12D/+^ pancreatic cells with *Muc4* overexpression secreted Activin A for fibroblast activation and recruitment (Figure 5C–F), and close contact with these fibroblasts further increased Activin A secretion (Figure 5G,H), which promotes spheroid and soft agar colony formation of *Kras* ^G12D/+^/*Muc4‐*overexpressing pancreatic cells (Figure 4E,F,I,J). This is entirely consistent with the previously reported observation of direct fibroblast‐PDAC tumor cell contact.^[^ ^33^ ^]^ However, compared to the PDAC study,^[^ ^33^ ^]^ our data showed that both PanIN cells and surrounding fibroblasts express Activin A (Figure 5I; Figure S17, Supporting Information), suggesting that pancreatic cells with *Kras* ^G12D/+^/*Muc4* overexpression may secrete a small amount of Activin A to initially activate/recruit fibroblasts. PanIN cell‐fibroblast contact may then enhance Activin A secretion from fibroblasts to further augment PanIN cell proliferation and malignancy as well as fibroblast activation. This concept was further validated by utilizing lentiviral shRNA to decrease Activin A expression from mPSCs for a coculture experiment in soft agar colony formation assay. The results, depicted in Figure 6F, demonstrated that mPSCs with reduced Activin A expression reduced capability in promoting the formation of soft agar colonies of double EpCAM/Muc4‐positive pancreatic cells, in comparison to PSCs with *LacZ* ^shRNA^, suggesting that the additional secretion of Activin A from fibroblasts in contact with *Kras* ^G12D/+^/ *Muc4* overexpressing pancreatic cells plays a crucial role in promoting PanIN formation. This finding is consistent with our previous study^[^ ^33^ ^]^ that direct contact between fibroblasts and tumor cells, mediated by homotypic ATP1A1 interaction, could induce the secretion of Activin A from fibroblasts. However, it would be valuable to further investigate the interplay between Kras^G12D^ and Muc4 overexpression in triggering Activin A secretion.

Given that the majority of PanIN cells with *Kras* ^G12D/+^ and *Muc4* overexpression expressed Activin mRNA, in contrast to normal ductal and acinar cells (Figure 5I), it is expected that oncogenic *Kras* ^G12D/+^ alone does not directly upregulate Activin A. The expression of Activin A is known to be regulated by the NF‐κB pathway, but oncogenic KRAS alone is insufficient to activate NF‐κB. However, pancreatic tumor cells driven by oncogenic KRAS exhibit constitutive NF‐κB activation,^[^ ^42^ ^]^ suggesting that additional conditions or factors are required for NF‐κB activation in the context of oncogenic KRAS. Therefore, it is plausible that oncogenic KRAS can collaborate with MUC4 to activate NF‐κB signaling, leading to subsequent Activin A expression. Furthermore, considering the observation that patients with dual high expression levels of *MUC4* and *INHBA* demonstrated a worse prognosis compared to the other group (Figure S18E, Supporting Information), it is important to investigate the mechanistic aspects of the molecular network involving in the crosstalk between oncogenic KRAS, MUC4, and Activin A. Such investigations could provide valuable insights for the development of novel treatment strategies for PDAC.

The critical role of Activin A in regulating the inflammatory response and immunity has been well established.^[^ ^43^ ^]^ Numerous studies have demonstrated the significant involvement of immune cell infiltration in pancreatic tumorigenesis.^[^ ^26^, ^44^ ^]^ However, it is worth noting that immune cells are relatively scarce in the early PanIN stage (Figure S13B,H; Figure S14, Supporting Information), indicating that their impact on PanIN formation may manifest at a later stage. Considering that PanIN represents the initial stage of PDAC development, investigating the reciprocal interactions among Activin A, oncogenic KRAS, and MUC4 within the context of the interplay between PanIN cells, fibroblasts, and immune cells holds great potential for uncovering therapeutic opportunities. Further exploration in this aspect is warranted.

In sum, the results described herein provide a significant advance in understanding how oncogenic KRAS initiates the earliest PanIN, the precursor of PDAC, in pancreas. The interplay between oncogenic *Kras* ^G12D/+^‐mediated genetic *Muc4* alterations and induced fibroblast activation/recruitment via Activin A appears to be essential for PanIN initiation as well as PDAC formation,^[^ ^33^, ^36^ ^]^ providing potential opportunities for early diagnostic and management strategies of PDAC.

## 4 Experimental Section

### Ethics Statement and Human specimens

Human pancreatic specimens with early lesions were obtained from two donors (Table S1, Supporting Information) with normal HbA1c, amylase, and lipase levels.^[^ ^45^ ^]^ Tissue arrays, PA483e and HPan060SC02, with normal pancreas tissues, normal pancreas tissues adjacent to the tumor (NAT), and PDAC tumors were purchased from US Biomax Inc. For MUC4 IHC staining analysis, it was utilized 13 normal pancreas and NAT tissues, which do not contain PanINs, as well as 44 stage I/II PDAC samples. The relevant patient information had been included in Figure S5A (Supporting Information). Patients’ serum collection for Activin A analysis was described previously.^[^ ^46^ ^]^ Clinical demographic data in pancreatic ductal adenocarcinoma (PDAC) patients and high‐risk controls (HRC) were shown in Table S3 (Supporting Information). The High‐Risk Controls (HRCs) are individuals with a family history of PDAC who remained free from pancreatic malignancies throughout a follow‐up period exceeding 2 years. These individuals actively participated in the pancreatic cancer screening program conducted at the National Taiwan University Hospital between January 2005 and December 2015. As part of the program, the HRCs underwent a comprehensive evaluation, including a detailed history assessment, physical examination, collection of family history information, magnetic resonance imaging/magnetic resonance cholangiopancreatography (MRI/MRCP) examination, and blood testing. The application for the collection and use of human samples was submitted to the National Taiwan University Hospital (NTUH), Taipei, Taiwan, and approved by the Institutional Review Board of the NTUH (201303029RINC) and NTUH (201701015RINA).

### Animal Protocol and Transgenic Mice

All animal experiments were approved by the Institutional Animal Care and Utilization Committee of Academia Sinica, Taipei, Taiwan (IACUC#10‐04‐065). All mice were maintained in an SPF (specific pathogen‐free) animal facility at 20±2 °C with a 12/12 h light/dark cycle and had free access to water and a standard laboratory chow diet. *LSL‐Kras^G12D^* mice^[^ ^6^ ^]^ and *Elastase‐CreER* mice^[^ ^47^ ^]^ were obtained from Dr. Chia‐Ning Shen (Academia Sinica, Taipei, Taiwan). *Pdx‐1‐Cre* mice, a kind gift from Dr. Ting‐Fen Tsai (National Yang Ming Chiao Tung University, Taipei, Taiwan) and Dr. Kuang‐Hung Cheng (National Sun Yat‐sen University, Kaohsiung, Taiwan) under material transfer agreements, were generously made available by Douglas A. Melton.^[^ ^48^ ^]^ *LSL‐Kras^G12D^* mice, which were functionally heterozygous for the wild‐type allele (*Kras^+/−^*), were crossed with *Pdx‐1‐Cre* mice to produce *Pdx1‐Cre*; *LSL‐Kras^G12D/+^* (KC) mice that develop ductal lesions identical to the stages of PanIN and *Pdx1‐Cre*; *LSL‐Kras^+/+^* (control) mice. For investigating early‐stage PanIN formation in the adult mouse model, *Elastase*‐*Cre*ER; *LSL*‐*Kras* ^G12D/+^ (EK) mice were injected with tamoxifen (Sigma–Aldrich #T5648) at the age 6‐week‐old (2 mg injection^−1^, three injections in 1 week to induce *Cre*‐mediated recombination), and duct lesions were allowed to develop for the next 10 to 12 weeks.

### Immunofluorescence Staining for Whole Pancreas 3D Histology

After using 0.9% NaCl buffer to remove blood through cardiac perfusion, pancreatic blood vessels were labeled with Alexa Fluor 488 conjugate wheat germ agglutinin (Invitrogen #W11261) at 2‐fold body volume with the concentration of 16 mg mL^−1^ followed by 4% paraformaldehyde (PFA) perfusion for fixation. For 2‐ and 4‐ week‐old KC and control mice, the pancreas was harvested and post‐fixed with fresh 4% PFA, 30 min at RT. Before antibody staining, tissues were treated with the blocking solution (2% Triton X‐100, 10% normal goat serum, and 0.02% sodium azide in PBS) at 4 °C overnight. For an 18‐week‐old EK mouse, after blood vessel labeling, the pancreas was harvested and post‐fixed in 4% PFA solution for 40 min at 15 °C. Vibratome sections of the fixed tissue (≈500 µm) were then immersed in 2% Triton X‐100 solution for 2 h at 15 °C for permeabilization. Tissues were incubated sequentially with the 300‐fold dilution of primary antibodies (rabbit anti‐CK19 (Abcam #ab133496) and mouse anti‐αSMA (Abcam #ab7817)) at 4 °C for 2 days, and 300‐fold dilution of secondary antibodies (goat anti‐rabbit Alexa fluor 555 (Invitrogen #A32723) and donkey anti‐mouse Alexa fluor 647 (Invitrogen #A31571) at 4 °C for 1 day, and 1 µg mL^−1^ of nuclei dyes (DAPI, Invitrogen #D1306) at 4 °C for 30 min. After immunostaining, an optical clearing solution (RC1.52, SunJin Lab) was applied to make the sample transparent, which enhanced imaging depth by confocal microscope acquisition.

### Whole Pancreas Mounting

A plastic spacer (iSpacer, SunJin Lab) was used, stacked on 24 × 40 mm cover glass, to make the different volume containers. The samples were loaded on these hand‐made containers and filled full reflection index matching solution (RapiCler1.52, SunJin Lab #RC152002). The top of the container was sealed with another cover glass. Finally, the whole pancreas was mounted in a hand‐made coverslip container and could be observed by a confocal microscope.

### Microscope and Imaging Acquisition

Whole pancreas imaging was scanned by Zeiss LSM880 Confocal microscope (Carl Zeiss, Germany), which was equipped with 405, 488, 561, a 633 nm lasers, and four PMT detector. 5 µm optical section scanning (10x Plan‐Apochromat objective was used, N.A. 0.45, Carl Zeiss, Germany) to reconstruct the 3D distribution of antibody‐indicated protein markers. It was also acquired the high‐quality imaging of pancreatic lesions, which used a 2.5 µm optical section and 25x objective (LD LCI Plan‐Apochromat objective, N.A. 0.8, Carl Zeiss, Germany)

### Image Segmentation and Quantification

Each optical section of 3D imaging was projected on one flat for physically mapping lesion localization. The localization of each pancreatic lesion was doubly checked with the original imaging, and the distribution of total lesions was constructed on a whole pancreas model.

### Immunohistochemistry and Alcin Blue Staining for 2D Pancreatic Histology

Immunohistochemistry (IHC) was performed on a BOND‐MAX fully automated IHC staining system (Leica). 4 µm paraffin sections were baked, deparaffinized, and stained in this instrument. Slides for mouse Muc4, Muc5ac, αSMA, CD45, and human MUC5AC staining were antigen‐retrieved at PH 9, 95 °C, 30 min. Slides for mouse Muc1 and Muc6 were antigen‐retrieved at PH 9, 100 °C, 20 min. Slides for human MUC4 and Ki67 staining antigen retrieved at PH 6, 95 °C, 30 min. Dilutions and incubations of antibodies were 1:200, 60 min at room temperature (RT) for mouse anti‐Muc4 antibody (Thermo Fisher Scientific #35‐4900); 1:1000, 30 min at RT for mouse anti‐Muc5ac/MUC5AC antibody (Genetex #GTX11335); 1:50, 30 min at RT for mouse anti‐Muc1 (MRQ‐17) antibody (Cell MARQUE #290M‐16); 1:200, 30 min at RT for mouse anti‐Muc6 (MRQ‐20) antibody (MARQUE #293M‐96); 1:200, 60 min at RT for mouse anti‐αSMA (Novus #NB‐600‐531); 1:100, 60 min at RT for rat anti‐CD45 (eBioscience #14‐0452‐85); 1:100, 60 min at RT for rabbit anti‐MUC4 antibody (Abcam #ab183320); 1:1000, 30 min at RT for rabbit anti‐Ki67 antibody (Abcam #ab15580), followed by 10 min incubation with HRP‐conjugated secondary antibody (Bond refine systems, Leica). For mouse Muc4, Muc5ac, and αSMA staining, N‐Histofine MOUSESTAIN KIT (Nichirei Corporation #414321F) for specific mouse antibody detection was used. To facilitate mouse Muc1 and Muc6 staining, the mouse‐to‐mouse blocking reagent (ScyTek #MTM‐IFU) prior to primary antibody staining was employed. After primary antibody staining, Rabbit anti‐mouse IgG IgM and polymer (Goat anti‐Rabbit IgG conjugated HRP) for the staining process were utilized. Later, slides were stained with a DAB detection kit (Bond refine systems, Leica) for 5 min and counterstained with hematoxylin (Bond refine systems, Leica) for 7 min. All slides were mounted with coverlid and scanned at 40x magnification by Aperio GT450 (Leica). For Ki67 quantification, all PanIN regions per mouse, at least 10 normal ducts per mouse, and 30 randomly selected ROIs (1 mm^2^) of acinar regions per mouse were selected and quantified with a positive cells detection algorithm from QuPath. In this algorithm, the threshold is determined by the Nucleus: DAB OD mean measurements, and a threshold of 0.1 was performed to count Ki67 positive cells. Statistical data were presented as the percentage of positive cells.

For determining the expression level of Muc4 by IHC with anti‐Muc4 antibody, each 400x high‐power field of PanIN and normal duct regions were outlined by pen tools and analyzed by the positive pixel count v9 algorithm of Aperio ImageScope software (Aperio, USA) to calculate the average percentage of 1+, 2+, and 3+ cells. H‐score was assigned by [1 x (% of 1+ cells) + 2 x (% of 2+ cells) + 3 x (% of 3+ cells)]. At least 10 normal ducts per mouse were served as the control. For total mucin measurement, the pancreas slices (4 µm) from 4‐week‐old control and KC mice were stained with Alcian Blue, pH 2.5. To determine the positive ratio, two independent individuals counted the number of early PanIN with total mucin, Muc4, Muc5ac, Muc1, Muc6, αSMA, or CD45 in all PanINs.

### Microdissection of Early PanIN Cells

The captured PanIN cells and control acinar cells from 2‐week‐old KC mice were obtained through immunostaining of whole pancreas 3D histology samples. Using CK19 staining and 3D imaging, a ductal lesion was identified in a transparent pancreas specimen. The precise capture of pancreatic lesions and control acinar cells was achieved using a micro‐manipulator (MHW‐3, Narishige, Japan) equipped with a glass capillary connected to a PTFE tube and syringe, under the microscope. Subsequently, the captured cells were rinsed out from the glass capillary using ice‐cold PBS. Following this, they were transferred into ice‐cold PBS for a 30 min duration to eliminate the clearing reagent (RapidClear1.52), facilitating subsequent exome sequencing (WES) or RNA sequencing (RNA‐seq) analysis. The PanIN lesion typically exhibits a 3D structure with diameters ranging from 80 to 120 µm. For each PanIN, ≈300 to 1000 cells were captured for WES and RNA‐seq analysis.

### Whole Exome Genomic Variants Analysis

Total DNA was isolated from tissue samples using QIAamp DNA Mini Kit (QIAGEN). The quantity of DNA was measured by reading A260/280 ratios by Nanodrop. The DNA library was constructed using Ovation Ultralow Library Systems V2 (NuGEN Technologies, Redwood City, CA, USA). Exome capture was performed using SureSelect XT Mouse All Exon Kit (Agilent Technologies) following the vendor's recommended protocol, and sequencing was performed using the Illumina NovaSeq 6000 sequencing platform for a 150‐bp paired‐end run. About 20–500 million paired‐end reads of 150 bp length were obtained. Data yielded 3–70 G of sequence, representing ≈60–1400 times the size of the mouse all exome (49.6 Mb). Before alignment, the low‐quality reads were removed. After that, the cleaned paired‐end reads were produced. For the alignment step, BWA^[^ ^49^ ^]^ is utilized to perform mouse (GCRM38) reference genome alignment with the reads contained in paired FASTQ files. Variant calls can be generated with GATK Haplotype Caller,^[^ ^50^ ^]^ which examines the evidence for variation from reference. In order to identify mutations, present in PanIN cells, a comparison between the genomic data obtained from the PanIN region and the normal pancreatic region within the same KC mouse was conducted. Furthermore, the PanIN data with the genomic data from two normal pancreatic regions acquired from control mice was compared. To ensure the accuracy of this analysis, it was excluded all mouse‐specific single nucleotide polymorphisms (SNPs) by referring to the UCSC Genome Browser website (https://genome.ucsc.edu/cgi‐bin/hgTrackUi?db = mm10&g = strainSNPs).

### RNA‐Seq Analysis

Early PanIN cells and adjacent normal acinar cells in the same 2‐week‐old KC mouse were captured using manual microdissection for library construction using the Ovation SoLo RNA‐Seq System (NuGEN Technologies, San Carlos, CA, USA). Total RNA was subjected to cDNA synthesis and NGS library construction. The quality and the average length of sequence library for each sample were assessed using Bioanalyzer (Agilent Technologies, Santa Clara, CA, USA) and the DNA 1000 kit (Agilent Technologies). The indexed samples were pooled equimolarly and sequenced on Illumina HiSeq4000 (150 base, paired‐end reads) (Illumina, San Diego, CA, USA). For data analysis, the quantification of raw reads was processed using CLC Genomics Workbench v.10 software. Adaptor sequences and bases with low quality or ambiguity were trimmed. The quality screened reads were mapped to mouse (GRCm38) genome using CLC Genomics Workbench. The mapping parameters were the following: mismatch cost 2, insertion cost 3, deletion cost 3, length fraction of 0.5, and similarity fraction of 0.8. The expression values were calculated as RPKM (Reads Per Kilobases per Million). The differential gene expression between two samples is based on the fold change of RPKM value. The genes were further analyzed as described in Figure S3 (Supporting Information). Gene ontology (GO) enrichment was analyzed by GO‐TermFinder.^[^ ^51^ ^]^

### Plasmids

Lentiviral GFP‐MUC4/X plasmid (pLenti‐GFP‐MUC4/X) was constructed by inserting the cDNA of GFP‐MUC4/X at the EcoRV site of pLAS5w.Pbsd vector. GFP‐MUC4/X cDNA was obtained by digesting pCMV3‐MUC4‐C‐GFPSpark (Sino Biological #HG16066‐ACG) with KpnI (NEB) and XbaI (NEB) and filling the sticky end with Klenow (NEB). pLAS5w.Pbsd vector was obtained from National RNAi Core Facility (Taipei, Taiwan). The retroviral pBABE‐puro (#1764; RRID: Addgene_1764) plasmid and retroviral pBabe‐Kras G12D‐puro plasmid (#58 902; RRID: Addgene_58 902), encoding HA‐KRASG12D, were acquired from Addgene (USA). The lentiviral shRNA expression vectors of pLKO.1‐shLacZ (TRCN0000072223), shInhba #1 (TRCN0000067740) and shInhba #2 (TRCN0000324943) were from the National RNAi Core Facility (Taipei, Taiwan).

### Lentivirus and Retrovirus Production

For lentivirus production, human 293T cells were co‐transfected with lentiviral constructs, packaging plasmid pCMVΔ8.91, and envelope plasmid pMD.G expressing VSV‐G. Virus‐containing supernatant was collected 48 h post‐transfection. For retrovirus production, human 293GP2 cells were co‐transfected with retroviral constructs and plasmid pMD.G. Virus‐containing supernatant was collected 48 h post‐transfection.

### Isolation and Culture of Primary Pancreatic Cells

Mouse pancreatic acinar cells were isolated and cultured as described previously.^[^ ^52^ ^]^ Briefly, pancreases from 4‐week‐old male control or KC mice were mechanically and enzymatically digested with collagenase IA solution (1x HBSS containing 10 mM HEPES, 200 units mL^−1^ of Collagenase IA, and 0.25 mg mL^−1^ of soybean trypsin inhibitor) to obtain isolated acinar structures. Acinar cells were grown in a medium containing one volume of glucose‐free DMEM and one volume of F12 medium supplemented with 2.5% FBS, 1% penicillin/streptomycin mixture, 0.25 mg mL^−1^ of trypsin inhibitor, and 25 ng mL^−1^ of recombinant human epidermal growth factor (EGF) (Thermo Fisher Scientific #17 075 029). After culturing for one day, most of the cell debris was removed and the remaining cells were used for subsequent experiments. When using non‐collagen coated plates, the cells maintain the acinar phenotype in suspension conditions for 2 to 3 days. After a 4‐day culture period, duct‐like cells, including ADM and PanIN cells, from KC mice, were able to attach and grow on the non‐collagen coated plate. However, only a few ADM and duct cells from control mice showed such capability to attach and grow under the same conditions.

Isolation and culture of mouse pancreatic stellate cells (mPSC) were based on that described by Apte et al.^[^ ^53^ ^]^ with some modifications, as described below. The pancreas from a 4‐week‐old male control mouse was cut into pieces of less than 1 mm^3^ and underwent enzymatic digestion using Collagenase P (1 mg mL^−1^; Roche #11 213 873 001), Collagenase/Dispase (1 mg mL^−1^; Roche #11 097 113 001), and soybean trypsin inhibitor (0.1 mg mL^−1^; Thermo Fisher Scientific #17 075 029) in HBSS at 37 °C for 15 min with intermittent pipetting twice. After neutralization with DMEM/F12 medium (supplemented with 10% fetal calf serum and 10 µg mL^−1^ antibiotics/ antimycotics), the cell suspension was filtered through a 100 µm nylon mesh into a 50 mL centrifuge tube. The tube was centrifuged at 300 g for 3 min at 4 °C. After carefully aspirating the supernatant, the cell pellet was washed with 5 mL of GBSS + NaCl containing 0.3% BSA and centrifuged at 300 g for 3 min at 4 °C. The supernatant was cautiously removed via pipetting, and the cell pellet was thoroughly suspended with 8 mL of 28.7% Nycodenz in GBSS + NaCl solution. Then, 6 mL of GBSS + NaCl with 0.3% BSA was added on top of the cell suspension in Nycodenz, not disrupting the interface. The tube was centrifuged at 1400 g for 20 min at 4 °C. The thin white band just above the interface was collected using a 5 mL transfer pipette without disturbing the density gradient layers. Cells were washed with GBSS + NaCl containing 0.3% BSA, centrifuged at 300 g for 3 min at 4 °C, and resuspended in stellate cells medium (ScienCell Research Laboratories, inc.). The harvested cells were cultured in a 100 mm petri dish and incubated in a humidified atmosphere with 5% CO_2_ at 37 °C.

Mouse pancreatic islet cells from 4‐week‐old male mice were isolated and cultured as described previously^[^ ^54^ ^]^ with some modifications. Briefly, the pancreas was perfused via the common bile duct with 4 mL HBSS containing Collagenase V (Sigma‐Aldrich #C9263) (1.5 mg mL^−1^). The dilated pancreas was removed and digested at 2 mL of HBSS containing Collagenase V at 37 °C for 20 min. Islets were freed by gentle agitation, stopped enzyme reaction in RPMI 1640 culture medium containing 1% FBS, washed in HBSS, and purified on Histopaque 1077 (Sigma–Aldrich #10 771) gradients. Islets were transferred to 100 mm petri dish with RPMI 1640 culture medium supplemented with l‐glutamine (2 mM), penicillin (100 U mL^−1^), streptomycin (100 µg mL^−1^), and 10% fetal bovine serum. After culturing for 24 h, islet cells were collected and dispersed into single cells with trypsin for subsequent experiments.

### Cell Culture

Human 293T cells (#CRL‐3216) and human GP2‐293 cells (#631 458) were purchased from ATCC and TaKaRa, separately, and grown in high glucose DMEM medium supplemented with 10% FBS and antibiotics (penicillin/streptomycin). The non‐transformed human pancreatic ductal epithelial cells (HPDE)^[^ ^55^ ^]^ (provided by Dr. Kelvin K. Tsai, National Health Research Institutes, Taiwan) were generated from a 75‐year‐old male pancreatic specimen grown in keratinocyte serum‐free (KSF) medium supplemented with 0.2 ng mL^−1^ EGF and 30 µg mL^−1^ bovine pituitary extract (Invitrogen Life Technologies). Human primary pancreatic stellate cells (hPSC) (#3830) were obtained from ScienCell Research Laboratories, inc., and cultured in stellate cell growth medium (ScienCell Research Laboratories, inc.). All cells were maintained at 37 °C in a humidified atmosphere containing 5% CO_2_ and regularly checked for mycoplasma infection.

### Virus Infection and Stable Line Establishment

To ectopically express GFP or GFP‐MUC4/X in primary pancreatic acinar cells, it was isolated primary acinar cells from 4‐week‐old control or KC mice. After one day of culture, acinar cells were infected with 10 multiplicity of infection (MOI) of lentiviral GFP or lentiviral GFP‐MUC4/X overnight. After recovering from virus infection for one day, GFP‐positive cells using 1 µg mL^−1^ puromycin selection for two days were enriched. After this selection, the cells recovered from puromycin treatment for one day before using them for further experiments. Figure S7 (Supporting Information) shows that more than 80% of cells were GFP‐positive cells.

To establish HPDE cells with stable expression of retroviral or lentiviral genes, the cells with 10 MOI of retrovirus or lentivirus containing the relevant cDNA overnight were infected. Following recovery from virus infection for two days, it was selected cells with stable expression of the cDNA using 1 µg mL^−1^ puromycin (Thermo Fisher Scientific) and 1 µg mL^−1^ blasticidin (Thermo Fisher Scientific) for 14 days. Following this selection, the cells recovered from puromycin and blasticidin treatment for 7 days and were checked for protein expression levels using Western blot analysis (Figure S9A, Supporting Information) before using them for further experiments.

To establish mPSCs cells with stable expression of lentiviral shRNA, an overnight infection of mPSCs with 10 MOI of the designated lentiviral shRNA was performed. After allowing the cells to recover from the virus infection for one day, the cells that expressed the lentiviral shRNA by applying puromycin selection at a concentration of 1 µg mL^−1^ for a duration of three days was selectively enriched. Following a two‐day recovery period, the cells were then subjected to Western blot analysis and coculture experiments (Figure 6F).

### Cell Cluster Isolation and Immunofluorescence (IF) Staining

Pancreases were obtained from both control and KC mice at 4 weeks of age. The pancreas was then cut into small pieces of less than 1 mm^3^ and subjected to enzymatic digestion using Collagenase P (1 mg mL^−1^; Roche 11 213 873 001) and soybean trypsin inhibitor (0.1 mg mL^−1^; Thermo Fisher Scientific #17 075 029) in HBSS containing Ca^2+^ and Mg^2+^ at 37 °C for 25 min with intermittent pipetting. The tissue fragments in the resulting mixture were neutralized with DMEM/F12 medium supplemented with 10% fetal calf serum and 10 µg mL^−1^ antibiotics/antimycotics before being transferred to a mechanical dissociator C tube, where they underwent dissociation twice with program A.01 (one cycle = 25 s). The resulting homogenate was passed through a 40 µm cell strainer and centrifuged at room temperature at 300 x g for 5 min. After washing the pellet with PBS, the dissociated cells were stained with Mouse anti‐EpCAM antibody (1:200; eBioscience #145791‐85) and Rabbit anti‐PDGFRα antibody (1:200; Abcam # ab203491) for 1 h at 4 °C. The cells were then incubated with secondary antibodies (1:200; goat anti‐rat Alexa Fluor 488 (InvitrogenTM #A‐11006) and goat anti‐mouse Alexa fluor 647 (InvitrogenTM #A32728) and eFluor 780 viability dye (eBioscience #65‐0865‐14) for fluorescence‐activated cell sorting (FACS). Prior to FACS analysis, the cluster cells were filtered through a tube with a 35 µm filter. The cluster cells were sorted by gating them based on the width (W) parameter.

For IF co‐staining, cell clusters were plated on poly‐L‐lysine‐coated glass slides and fixed with 4% paraformaldehyde. After permeabilization with 0.5% TritonX‐100 and blocking with 1% goat serum, cells were subjected to IF staining with rabbit anti‐CK19 antibody (1:100; Genetex #GTX112666) at 4 °C overnight. After washing with PBST (PBS + 0.1% Tween20), cells were incubated with secondary antibodies (Goat anti‐rabbit Alexa fluor 594 (Invitrogen # A‐11012)) for 1 h at room temperature. After washing with PBST, cells were incubated with αSMA‐Alexa fluor 488 (1:100; Thermo Fisher Scientific #53‐9760‐82) for 1 h at room temperature. The nucleus was stained with DAPI.

### Cyst Formation Analysis and IF Staining of Cyst

For cyst formation analysis, medium containing 50% Corning Matrigel Growth Factor Reduced (GFR) Basement Membrane Matrix (Merck# CLS356252) was added to a 96‐well dish and plated at room temperature for 10 min to allow the Matrigel to solidify. Cells in suspension were mixed and grown in culture medium containing 2% Matrigel. After 14 days, Cysts were stained with Hoechst33342 (Sigma–Aldrich #B2261) and analyzed by fluorescence microscope (Olympus IX71 with DP70 camera).

For IF staining of the cyst, ice‐cold PBS–EDTA was added to detach Matrigel from the bottom and shaken gently for 15 min at 4 °C. The solution was transferred to a microtube and gently shaken until Matrigel had been dissolved completely. Cysts were centrifuged at 2000 rpm for 1 min and applied onto poly‐L‐lysine‐coated coverslips for IF co‐staining of αSMA and CK19.

### Spheroid Formation Analysis and Soft Agar Colony Formation Analysis

For spheroid formation analysis, 1000 primary pancreatic cells or 1000 HPDE cells were cocultured with or without 2000 PSCs in a 96‐well ultra‐low attached dish in a humidified 37 °C incubator for 14 days. The number of spheres with the green signal with a diameter ≥ 100 µm was calculated under fluorescence microscopy (Olympus IX71 with DP70 camera).

For soft agar colony formation analysis, 1000 primary pancreatic cells or 1000 HPDE cells were cocultured with or without 2000 PSCs and seeded in a layer of 0.35% agar/complete growth medium over a layer of 0.5% agar/complete growth medium in a 96‐well plate in a humidified 37 °C incubator for 14 days or 28 days, respectively. The number of spheres with the green signal with a diameter ≥ 50 µm was calculated under fluorescence microscopy (Olympus IX71 with DP70 camera).

### Chemotaxis Analysis

Primary mPSCs and hPSC were labeled with CellTracker Green CMFDA dye (Invitrogen # C2925) at the concentration of 1 μ mL^−1^ for 10 mins before experiments. Conditional media from indicated cells or media containing the indicated concentration of recombinant Activin A were applied for PSC μ‐Slide Chemotaxis (ibidi #80326) analysis.^[^ ^56^ ^]^ For Activin A neutralization experiment, conditional media were preincubated with 4 µg mL^−1^ anti‐activin A antibody (Biolegend # 693 604) at 37 °C for 30 min before chemotaxis analysis. After 24 h, the PSC chemotaxis effect was examined by fluorescence microscope (Olympus IX71 with DP70 camera) and calculated by Imaris software.

### Quantitative Real‐Time PCR

Total RNAs were extracted with TRIzol reagent (Invitrogen) and reversely transcribed with Transcriptor first strand cDNA synthesis kit (Roche #0 489 703 0001). To quantify specific gene expression, the quantitative real‐time RT–PCR was performed using KAPA SYBR FAST qPCR Kit (KAPA Biosystems) as manufacturer's instruction and analyzed on a Step One Plus Real‐Time PCR system (Applied Biosystems, Life Technologies). GAPDH was used as an internal control for gene expression. All primers were listed in Table S2.

### Cytokines Array Analysis and Activin a Quantification

Conditional media from the cell clusters isolated from 4‐week‐old control or KC mice were used for mouse cytokines analysis using RayBio Mouse Biotin‐Label Based Antibody Array (Mouse L‐308 Array, Glass Slide). For mouse Activin A quantification, Activin A in conditional media or serum was detected using the Human/Mouse/Rat Activin A Quantikine ELISA Kit (R&D Systems #DAC00B). For human Activin A quantification, Activin A in HPDE conditional media was detected using Human Activin a DuoSet ELISA Kit (R&D Systems #DY338). The amount of Activin A was normalized to the total protein in conditional media or sera and represented as pg mg^−1^.

### 3D/2D Integrative Histology for Mouse/Human Pancreatic Specimens

When PanIN lesions were detected in a 350 µm tissue section via 3D imaging, the section was processed by dehydration, embedding, and microtome sectioning to generate 4 µm paraffin slices for H&E staining (Leica Autostainer XL). H&E‐stained specimens were examined using the same Zeiss microscope for a side‐by‐side comparison of the 3D fluorescence and 2D H&E micrographs to confirm the PanIN lesion. Once confirmed, multiplex signals from the same microenvironment were further acquired from adjacent sections via standard immunohistochemistry (4 µm sections; Bond Polymer detection kit, Leica Biosystems #DS9800/DS9390) and 3D histology (350 µm sections) as described previously.^[^ ^45^ ^]^

### RNA and Protein Mixed Multiplex Fluorescence IHC

To assess the expression of RNA and protein in a mixed multiplex staining assay, an opal 4 color fIHC kit (PerkinElmer #NEL820001KT), and RNA scope multiplex fluorescent V2 assay (Advanced cell diagnostics #323 120) were applied based on the reported procedure.^[^ ^57^ ^]^ For RNA‐fluorescence in situ hybridization, 5 µm slices of PPFE tissue were deparaffinized in xylene and rehydrated through ethanol and water. Subsequently, the tissue slices were subjected to a boiling temperature using a hot plate for 15 min in RNA scope target retrieval reagent, followed by treatment with protease plus reagent at 40 °C for 30 min in the HybEZ hybridization oven (Advanced cell diagnostics, #241000ACD). The slices were then hybridized with the target probe, preamplifier, amplifier, and HRP‐conjugated probe, and the resulting fluorescence was visualized using Opal 570 reagent (diluted 1:750). Subsequently, fluorescent IHC staining of proteins was performed using the BOND Max autostainer (Leica). The primary antibodies were incubated at room temperature, followed by incubation with HRP‐conjugated secondary antibodies using the Bond Polymer Refine Detection Kit (Leica). The signal was detected using Opal 520 and 690 reagents (diluted 1:500). In the mouse study, the antibodies used were rabbit anti‐CK19 (diluted 1:400, 1 h; Abcam # ab133496) and mouse anti‐α‐SMA (diluted 1:100, 1 h; Biolegend #614852). In human study, the antibodies used were mouse anti‐CK19 (diluted 1:5000, 15 min; GeneTex #GTX27755) and mouse anti‐α‐SMA (diluted 1:5000, 15 min; Abcam #ab7817). DAPI staining was performed to visualize the nuclei, and slides were mounted with ProLong Gold antifade reagent (Thermo Fisher Scientific, P36930). Image acquisition was carried out using Zeiss LSM 880 confocal microscopes (Carl Zeiss, Jena, Germany) equipped with a ×63 Plan‐Apochromat and a ×100 alpha Plan‐Apochromat lenses. Zen 2.3 software (Carl Zeiss) and AimImageBrowser software (Carl Zeiss) were utilized for noise reduction (Gaussian filter; kernel size 3 × 3), signal segmentation, pseudo‐coloring, and exporting of the images.

### Western Blotting Analysis

Cells were sonicated or homogenized in RIPA buffer (20 mM Tris‐HCl, pH 7.5, 150 mM NaCl, 1 mM EDTA, 1% NP‐40, 0.1% SDS, and 1% sodium deoxycholate). After being clarified by centrifugation at 12 000 g for 15 min at 4 °C, the supernatant was collected for Bradford protein assay. Western blotting was performed as previously reported.^[^ ^58^ ^]^ Briefly, equal molarity of protein extracts was loaded and separated in an SDS–PAGE, and transferred to a PVDF membrane. After being blocked with 5% skimmed milk at RT for 1 h, the membrane was incubated with primary antibodies (rabbit anti‐MUC4 (1:1000; Thermo Fisher Scientific #35‐4900), ribbit anti‐GFP (1:10 000; GeneTex #GTX113617), rabbit anti‐KRAS G12D (1:1000; Cell Signaling Technology #14 429), rabbit anti‐KRAS (1:2000; Cell Signaling Technology #67 648), rabbit anti‐Activin A (1:1000; GeneTex #GTX108405), mouse anti‐GAPDH (1:10 000; GeneTex #GTX627408) at 4 ˚C overnight and treated with Goat Anti‐Rabbit IgG (HRP) (1:3000; GeneTex #GTX213110‐01) and Goat Anti‐Mouse IgG (HRP) (1:3000; GeneTex #GTX213111‐01) antibodies at room temperature for 1 h. Chemiluminescent detection of the horseradish peroxidase reaction was performed using Immobilon Forte Western HRP substrate (Merck #WBLUF0500) according to the manufacturer's instruction and filmed by ChemiDoc MP Imaging System (Biorad).

### Statistics Analysis

A two‐tailed Student's *t*‐test was used for immunohistochemistry analysis, cyst formation, spheroid formation analysis, soft agar colony formation, μ‐slide chemotaxis analysis, qPCR analysis, and Activin A quantification. ^*^, ^**^, ^***^, and ^****^ indicate *P* < 0.05, *P* < 0.01, *P* < 0.001, and *P* < 0.0001, respectively. N number and n number were shown in the figure legends. N indicates independent experiment, and n indicates cyst number or repeated assay. Survival curves were plotted using the Kaplan–Meier method and were then compared with the log‐rank test. The statistical analysis was performed using Excel and GraphPad Prism 9 (GraphPad Software, USA).

## Conflict of Interest

The authors declare no conflict of interest.

## Author Contributions

C.‐M.H. and W.‐H.L. are co‐corresponding authors. C.‐M.H. performed conceptualization, data curation, formal analysis, validation, investigation, visualization, methodology, wrote the original draft, wrote, reviewed, and edited, project administration, funding acquisition, and supervision. C.‐C.H. performed investigation, methodology, visualization, and formal analysis, and wrote the original draft. M.‐F.H. performed investigation, formal analysis, methodology, visualization, and validation. H.‐J. C. performed investigation, formal analysis, methodology, visualization, and validation. P.‐J.W. performed investigation, formal analysis, and validation. Y.‐I.C. performed the investigation and methodology. Y.‐M.J. performed the investigation and methodology. S.‐C.T. performed data curation and methodology. M.‐H.C. performed investigation and visualization. C.‐N. S., M.‐C.C., Y.‐T. C., and Y. W. T. provided resources. W.‐H.L. performed conceptualization, wrote the original draft, wrote, reviewed, and edited, project administration, funding acquisition, supervision.

## Supporting information

Supporting Information

## Data Availability

The mouse whole exome genomic variants and RNA‐seq data have been deposited in the Sequence Read Archive (SRA) under accession codes from SRR21619431 to SRR21619432, SRR21748899 to SRR21748931, and SRR24548379 to SRR24548380 within NCBI BioProject PRJNA881295.^[^ ^59^ ^]^
