# Supplementary material for: Oncogenic KRAS, Mucin 4, and Activin A‐Mediated Fibroblast Activation Cooperate for PanIN Initiation
Source: Adv Sci (Weinh). 2023 Nov 14;10(36):2301240. doi: 10.1002/advs.202301240 (PMC10754145; doi:10.1002/advs.202301240)
Supplement: Supplementary file 1 — Supporting Information [file ADVS-10-2301240-s001.pdf]

ADVANCED SCIENCE
Open Access

Supporting Information

for *Adv. Sci.*, DOI 10.1002/advs.202301240

Oncogenic KRAS, Mucin 4, and Activin A-Mediated Fibroblast Activation Cooperate for PanIN Initiation

Chun-Mei Hu, Chien-Chang Huang, Min-Fen Hsu, Hung-Jen Chien, Pei-Jung Wu, Yi-Ing Chen, Yung-Ming Jeng, Shiue-Cheng Tang, Mei-Hsin Chung, Chia-Ning Shen, Ming-Chu Chang, Yu-Ting Chang, Yu-Wen Tien and Wen-Hwa Lee**

Supporting Information

Oncogenic KRAS, Mucin 4, and Activin A-mediated Fibroblast Activation Cooperate for PanIN Initiation

Chun-Mei Hu*, Chien-Chang Huang, Min-Fen Hsu, Hung-Jen Chien, Pei-Jung Wu, Yi-Ing Chen, Yung-Ming Jeng, Shiue-Cheng Tang, Mei-Hsin Chung, Chia-Ning Shen, Ming-Chu Chang, Yu-Ting Chang[8], Yu-Wen Tien[7], Wen-Hwa Lee*.

*Corresponding authors: Chun-Mei Hu and Wen-Hwa Lee.
Email: CMHU1220@gate.sinica.edu.tw and whlee@uci.edu

This document includes:
Figure S1 to S17
Table S1 to S3

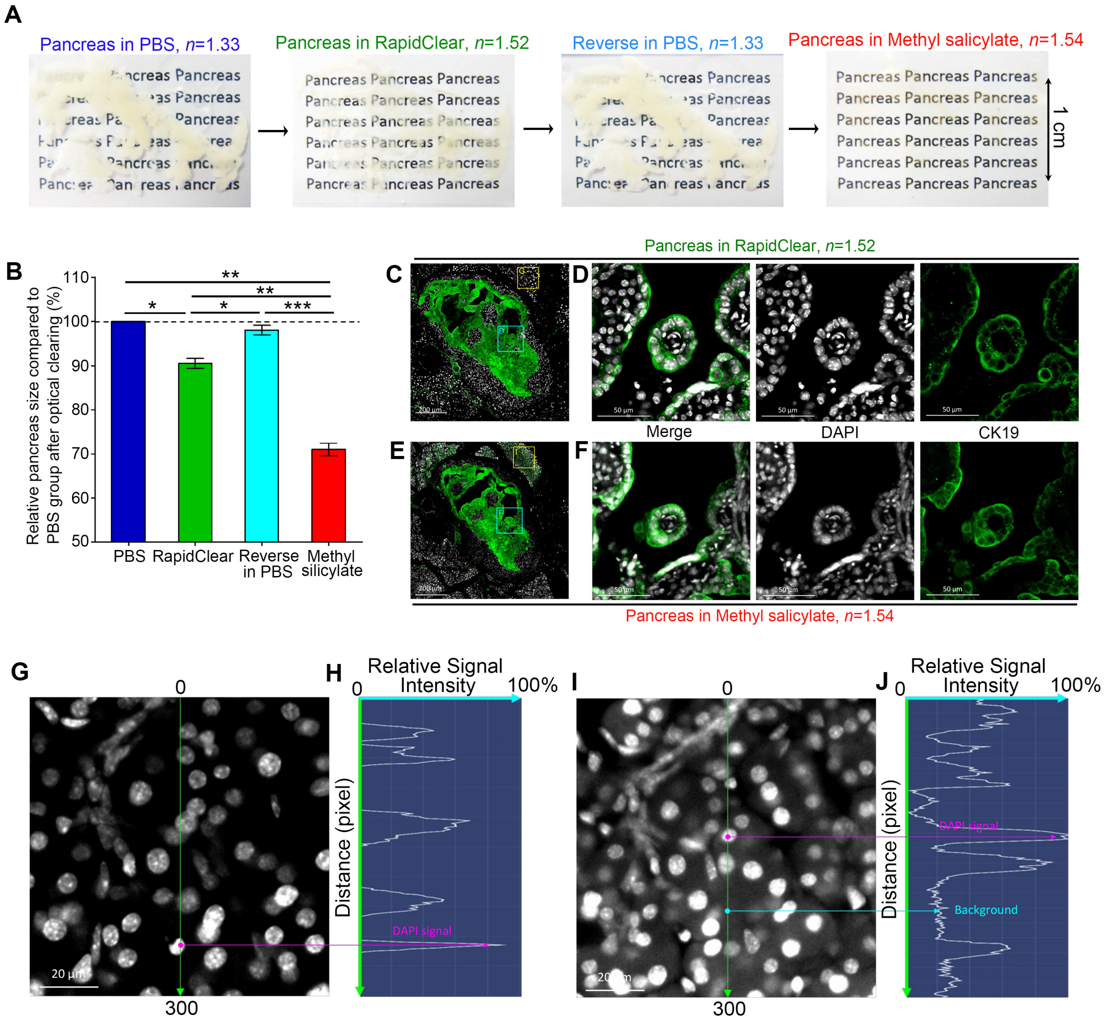

Figure S1. Comparative study between RapiClear and methyl salicylate for 3D whole tissue histology.

The mouse pancreas, which had been fixed, was sliced into sections of 350 μm thickness using a vibratome. To analyze tissue shrinkage, the pancreas sections were initially placed in PBS, and images were captured using an Olympus Stylus XZ-2 camera as a size reference. Subsequently, the sections were immersed in RapiClear at room temperature overnight. After the clearing process with RapiClear, the transparent sections were rinsed in room temperature PBS for 1 hour until they became opaque, effectively removing the RapiClear reagent. For the subsequent FLASH clearing, the pancreas sections underwent a gradual dehydration process using 30%, 50%, 75%, and 2×100% methanol in PBS. This was followed by clearing in 25%, 50%, 75%, and 2×100% methyl salicylate (Merck, cat. no. M6752) in methanol. Finally, the pancreas sections were immersed in 100% methyl salicylate for 30 minutes. At each step of the optical clearing and reversal process, photos of the pancreas sections were taken for quantification purposes. **A)** 350μm thick pancreas section shows different light penetration effects after being immersed in different refractive index solutions. The same text "Pancreas" placed at the bottom allows for evaluating the transparency of the pancreas section. *n*, refractive index. **B)** Tissue shrinkage is observed following immersion in a high-refractive-index solution

for optical clearing. The 350μm-thick pancreas section exhibits mild shrinkage when treated with the water-soluble clearing reagent, RapiClear. The dimensions of the tissue can be restored by rinsing out RapiClear with PBS. Moderate shrinkage is observed during methyl salicylate-based optical clearing. Three pancreas sections of equal thickness (350μm) were utilized for quantitative size measurement. The results are presented as a percentage of the tissue section size compared to the PBS reference. The data is expressed as means ± standard deviation. Statistical analysis was performed using a two-sided paired Student's t-test (*P < 0.05; **P < 0.01; ***P < 0.001). **C, E)** The 3-D projection images of the same PanIN treated with RapiClear reagent **(C)** and methyl salicylate **(E)**, respectively. Projection depth: 140 μm. **D, F)** Enlarged optical sections obtained from the identical region and depth in **C** and **E**. Tissue shrinkage can be observed in **F** from both the nucleus and epithelial signals. **G, I)** Enlarged adjacent normal pancreatic lobe after optical clearing. Relative signal analysis of the green line in **G** and **I** (300 pixels) is presented in **H** and **J)**. Magenta arrows show the signal from nucleus, cyan arrow in **J** indicates the background autofluorescence from acini.

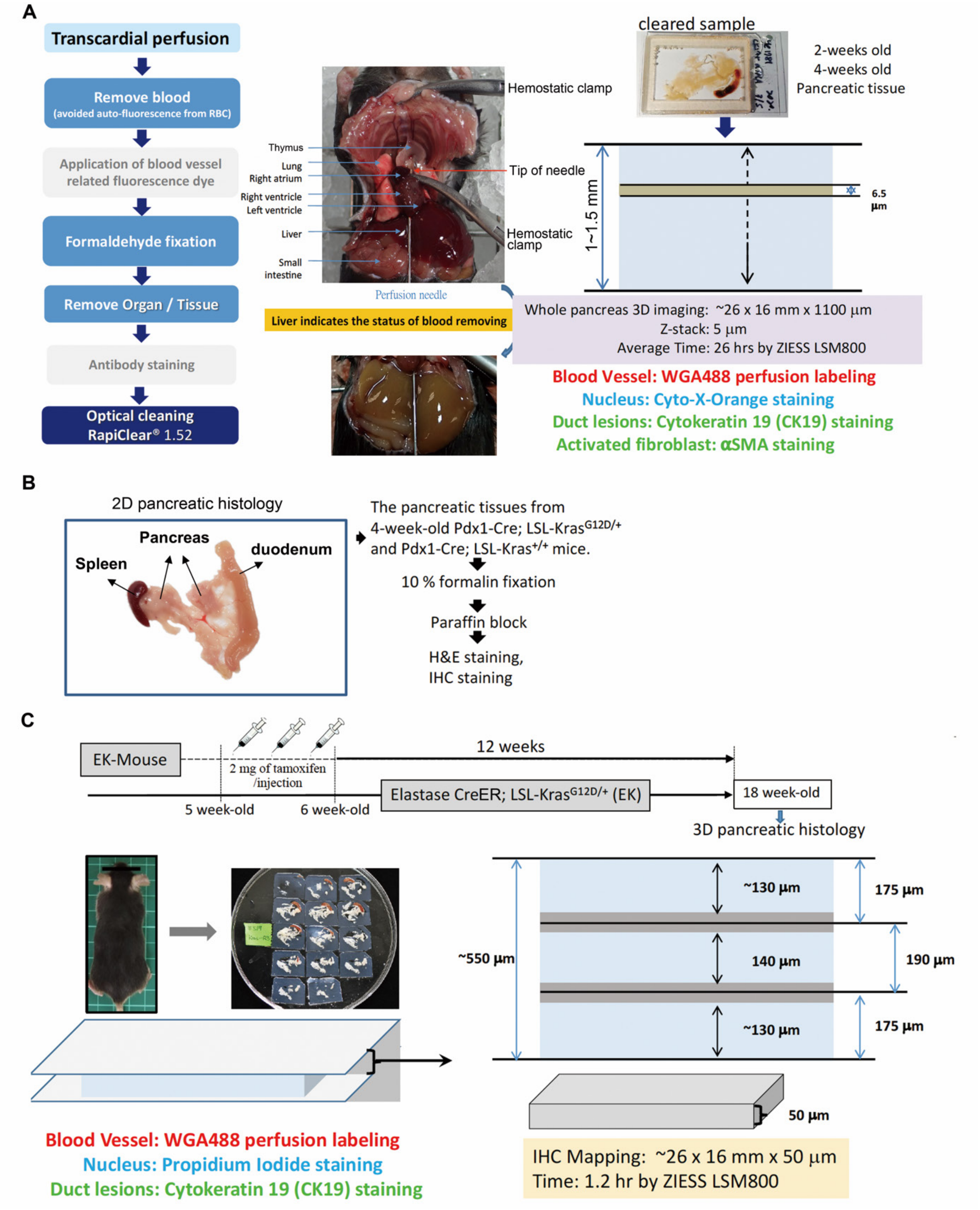

Figure S2. Experimental procedures are used for 3D and 2D pancreatic histology.
A) The whole pancreas 3D histology procedure for 2- and 4- week-old *Pdx1-Cre*; *LSL-Kras*$^{+/+}$ (control) and *Pdx1-Cre*; *LSL-Kras*$^{G12D/+}$ (KC) mice. **B)** The conventional approach of 2D pancreatic histology. **C)** The procedure of 3D pancreatic histology for *Elastase-CreER*; *LSL-Kras*$^{G12D/+}$ (EK) mouse model.

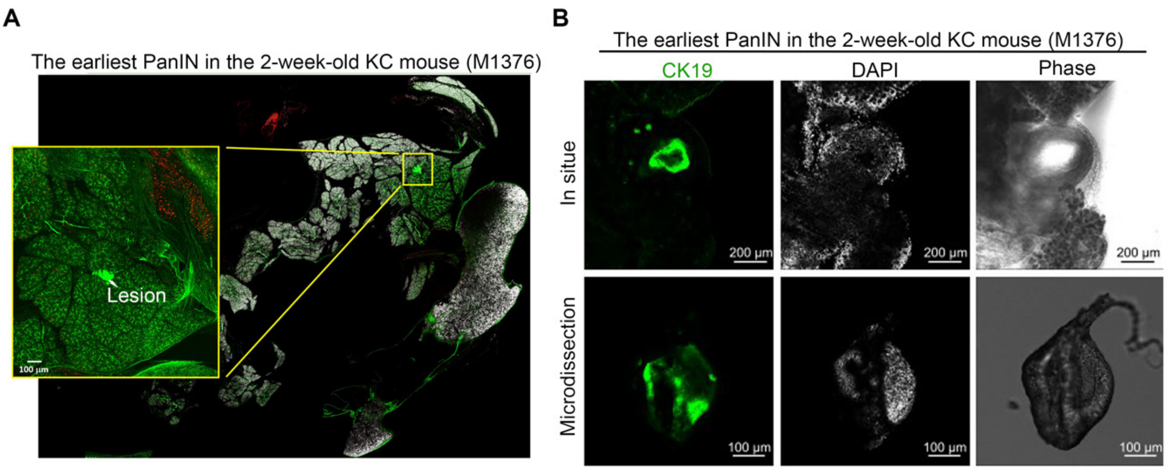

C

2-week-old KC mice

mouse number	Acinar region control	Lesion	Lesion number
1	M1546-Ctrl	M1546-Lesion 1	1
		M1546-Lesion 2	2
2	M1583-Ctrl	M1583-Lesion 1	3
3	M1608-Ctrl	M1608-Lesion 1	4
4	M1610-Ctrl	M1610-Lesion 1	5
5	M1542-Ctrl	M1542-Lesion 1	6
		M1542-Lesion 2	7
6	M1587-Ctrl	M1587-Lesion 1	8
		M1587-Lesion 2	9
7	M1531-Ctrl	M1531-Lesion 1	10
		M1531-Lesion 2	11
9	M1507-Ctrl	M1507-Lesion 1	12
		M1507-Lesion 2	13
		M1507-Lesion 3	14
10	M1503-Ctrl	M1503-Lesion 1	15
11	M1514-Ctrl	M1514-Lesion 1	16
		M1514-Lesion 1	17
12	M1520-Ctrl	M1520-Lesion 1	18
13	M1590-Ctrl	M1590-Lesion 1	19
		M1590-Lesion 2	20
		M1590-Lesion 3	21

2-week-old control mice

mouse number	Acinar region control		
1	M1523-Ctrl		
2	M1534-Ctrl		

Mouse SNPs database

The strains that have been sequenced and are in the variation catalog are:

129P2/OlaHsd	129S1/SvImJ	129S5SVEVBrd	A/J
AKR/J	BALB/CJ	BTBR	BUB/BnJ
C3H/HeH	C3H/HeJ	C57BL/10J	C57U/J
C57BR/cdJ	C57BL/J	C58/J	CAST/EiJ
CBA/J	DBA/1J	DBA/2J	FVB/NJ
I/LnJ	KK/HiJ	LEWES/EiJ	LP/J
MOLF/EiJ	NOD/ShiLtJ	NZB/B1NJ	NZO/HILtJ
NZW/LacJ	PWK/PhJ	RE/J	SEA/GnJ
SPRET/EiJ	ST/bJ	WSB/EiJ	ZALENDE/EiJ

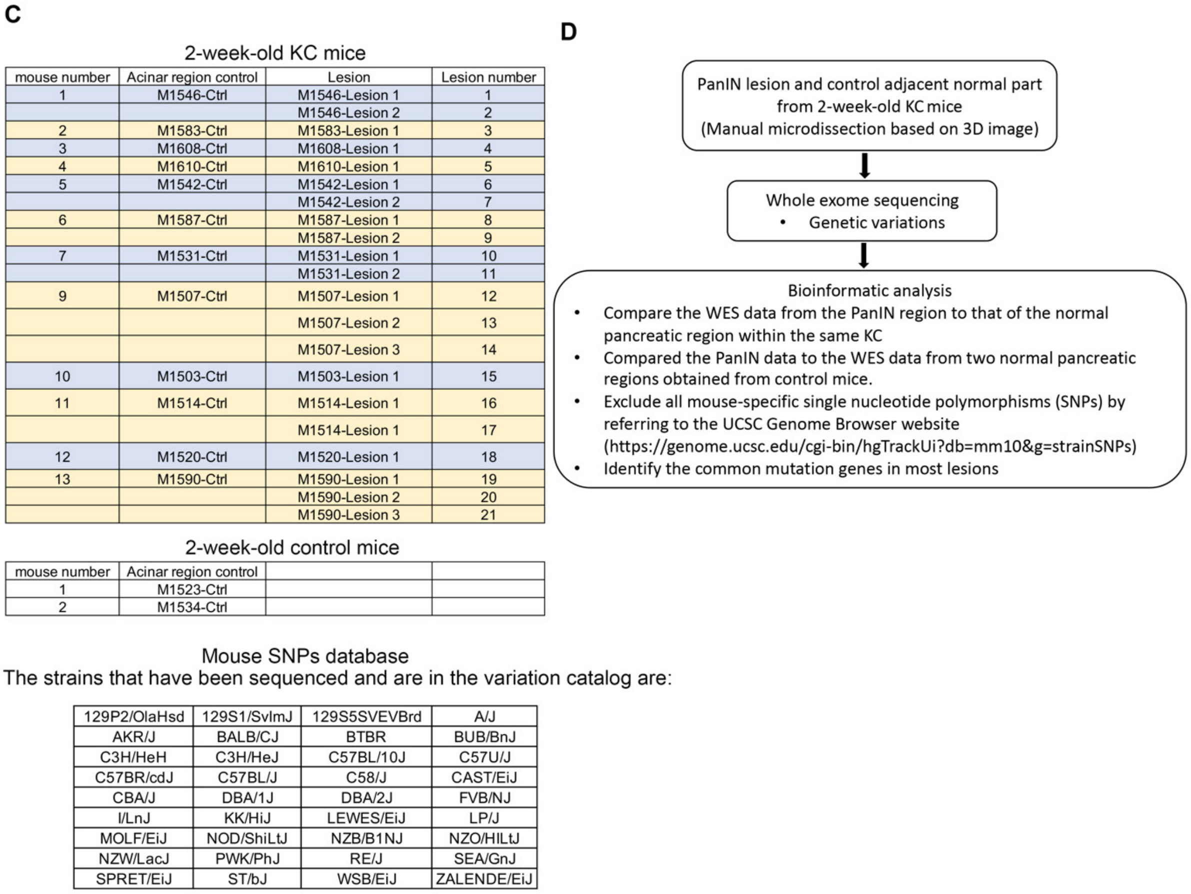

Figure S3. The earliest PanIN capture and genetic variants analysis.

A and B) Manual microdissection of the earliest PanIN lesion from a 2-week-old KC mouse. **(A)** The original location of PanIN is in the pancreas. **B)** Manual microdissection of PanIN. Green signal: lesion cells, CK19 staining. **C and D)** Procedures for genetic variations analysis. **(C)** Samples for whole-exome sequencing. Mouse SNPs database is from UCSC Genome Browser website (https://genome.ucsc.edu/cgi-bin/hgTrackUi?db=mm10&g=strainSNPs). They have used short-read sequencing to identify SNPs, indels, and structural variations relative to the C57BL/6J mouse reference genome. The strains that have been sequenced and are in our variation catalog are shown in the table. **D)** The flowchart for analysis.

A

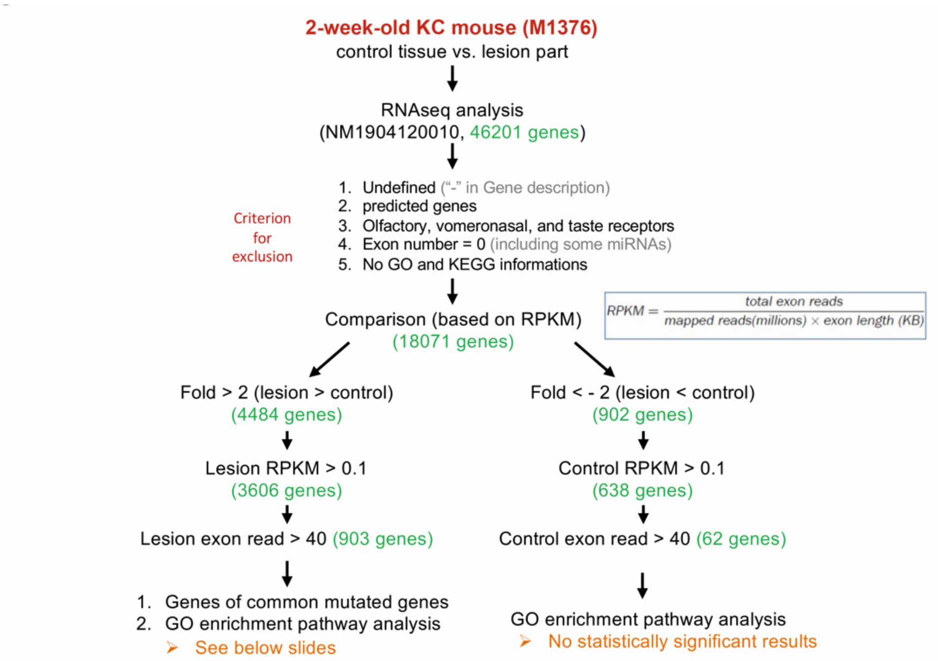

B

GO biological process complete	REFLIST (22265)	2 wk KC mice, lesion/Ctrl >2 (873)	Ratio (%)	Expected	over/ under	Fold Enrichment	raw P-value	FDR
regulation of neuronal synaptic plasticity (GO:0048168)	67	10	14.92537	2.63	+	3.81	6.18E-04	4.81E-02
positive regulation of JUN kinase activity (GO:0043507)	74	11	14.86486	2.9	+	3.79	3.46E-04	3.11E-02
negative regulation of ERK1 and ERK2 cascade (GO:0070373)	75	11	14.66667	2.94	+	3.74	3.83E-04	3.35E-02
protein autophosphorylation (GO:0046777)	197	25	12.69036	7.72	+	3.24	1.16E-06	3.83E-04
positive regulation of stress-activated MAPK cascade (GO:0032874)	167	19	11.37725	6.55	+	2.9	8.35E-05	1.17E-02
regulation of endothelial cell migration (GO:0010594)	160	18	11.25	6.27	+	2.87	1.45E-04	1.72E-02
positive regulation of stress-activated protein kinase signaling cascade (GO:0070304)	169	19	11.2426	6.63	+	2.87	9.63E-05	1.28E-02
regulation of stress-activated MAPK cascade (GO:0032872)	229	25	10.91703	8.98	+	2.78	1.29E-05	2.62E-03
regulation of stress-activated protein kinase signaling cascade (GO:0070302)	232	25	10.77586	9.1	+	2.75	1.58E-05	3.01E-03
positive regulation of MAP kinase activity (GO:0043406)	223	24	10.76233	8.74	+	2.74	2.36E-05	4.15E-03
regulation of peptidyl-serine phosphorylation (GO:0033135)	161	17	10.55901	6.31	+	2.69	4.29E-04	3.59E-02
cell cycle checkpoint (GO:0000075)	152	16	10.52632	5.96	+	2.68	6.45E-04	4.90E-02
regulation of JNK cascade (GO:0046328)	184	19	10.32609	7.21	+	2.63	2.63E-04	2.61E-02
positive regulation of protein serine/threonine kinase activity (GO:0071902)	284	29	10.21127	11.14	+	2.6	1.42E-05	2.74E-03
activation of protein kinase activity (GO:0032147)	245	25	10.20408	9.61	+	2.6	3.63E-05	5.67E-03
exocytosis (GO:0006887)	213	21	9.859155	8.35	+	2.51	3.47E-04	3.09E-02
positive regulation of DNA-binding transcription factor activity (GO:0051091)	246	24	9.756098	9.65	+	2.49	1.27E-04	1.57E-02
positive regulation of protein kinase activity (GO:0045860)	441	43	9.750567	17.29	+	2.49	2.14E-07	9.93E-05
regulation of synaptic plasticity (GO:0048167)	218	21	9.633028	8.55	+	2.46	4.02E-04	3.42E-02
regulation of epithelial cell migration (GO:0010632)	229	22	9.606987	8.98	+	2.45	2.90E-04	2.73E-02
positive regulation of kinase activity (GO:0033674)	512	49	9.570313	20.08	+	2.44	5.75E-08	3.49E-05
positive regulation of transferase activity (GO:0051347)	589	54	9.168081	23.09	+	2.34	4.31E-08	2.73E-05
regulation of MAP kinase activity (GO:0043405)	297	27	9.090909	11.65	+	2.32	1.60E-04	1.80E-02
regulation of cellular protein localization (GO:1903827)	558	49	8.781362	21.88	+	2.24	6.81E-07	2.62E-04
regulation of lipid metabolic process (GO:0019216)	346	29	8.381503	13.57	+	2.14	2.89E-04	2.74E-02
regulation of cell morphogenesis involved in differentiation (GO:0010769)	348	29	8.333333	13.64	+	2.13	3.07E-04	2.82E-02
negative regulation of protein modification process (GO:0031400)	574	47	8.188153	22.51	+	2.09	8.31E-06	1.99E-03
regulation of cellular component size (GO:0032535)	405	33	8.148148	15.88	+	2.08	1.85E-04	2.02E-02
DNA metabolic process (GO:0006259)	627	51	8.133971	24.58	+	2.07	3.01E-06	8.80E-04
regulation of actin cytoskeleton organization (GO:0032956)	360	29	8.055556	14.12	+	2.05	6.28E-04	4.86E-02
negative regulation of protein phosphorylation (GO:0001933)	412	33	8.009709	16.15	+	2.04	2.25E-04	2.31E-02

Figure S4. Gene expression profiles of the earliest PanIN lesion from a 2-week-old KC mouse.

Control pancreatic and PanIN cells were captured from a 2-week-old KC mouse by manual microdissection and subjected to RNA-seq analysis. **A)** The flowchart and criterion for RNA-seq analysis. **B)** GO enrichment pathway analysis.

–

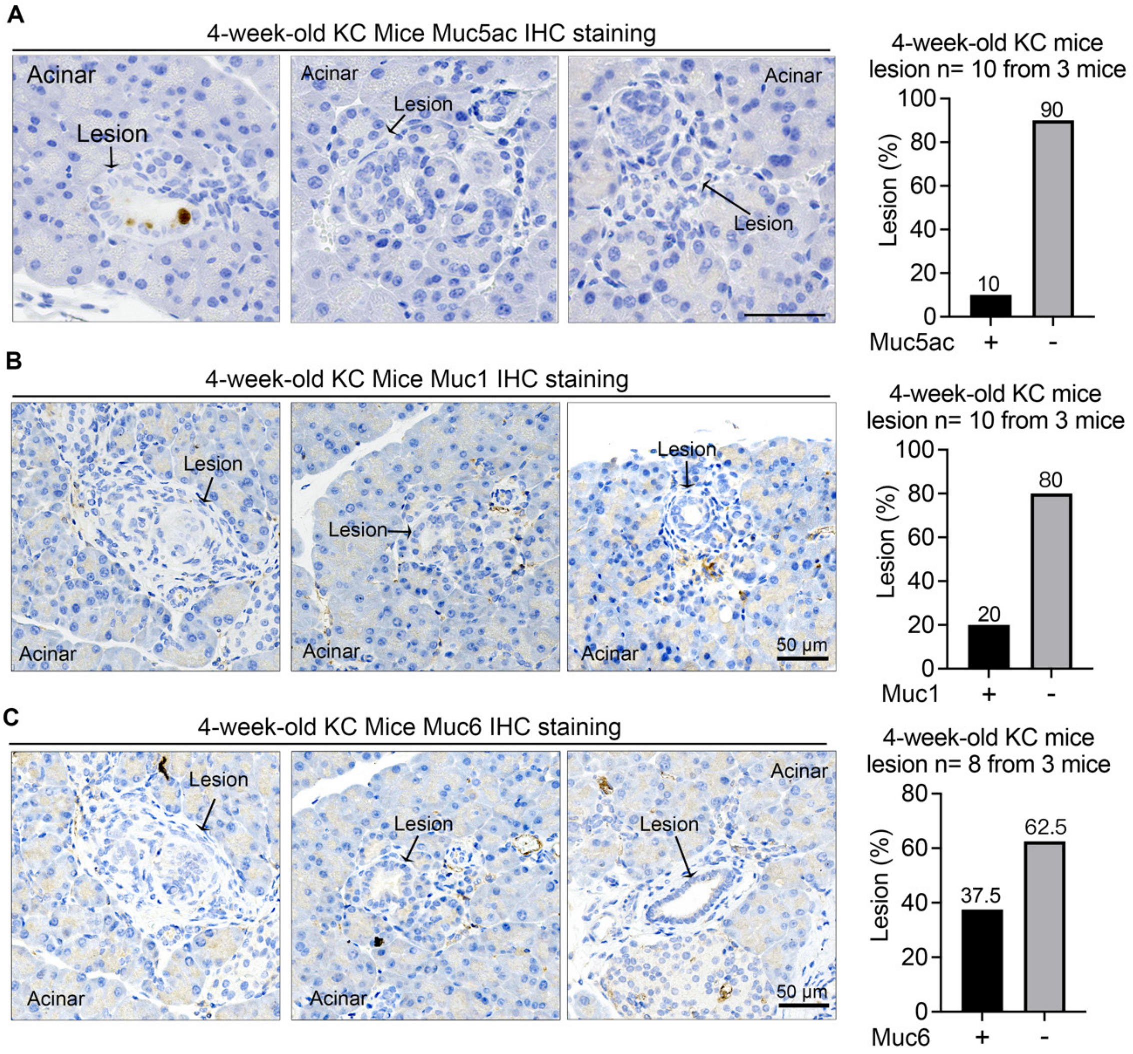

Figure S5. Only a minority of early PanIN cells in 4-week-old KC mice exhibited expression of Muc5ac, Muc1, and Muc6.

A-C) Immunohistochemical analysis using anti-Muc5ac antibody **(A)**, anti-Muc1 antibody **(B)**, and anti-Muc6 antibody **(C)** was conducted in 4-week-old KC mice. The left panels of A-C display representative IHC staining images for Muc5ac, Muc1, and Muc6. The right panels of A-C present quantification results for Muc5ac, Muc1, and Muc6 within the early PanIN cells. Bar, 50 μm.

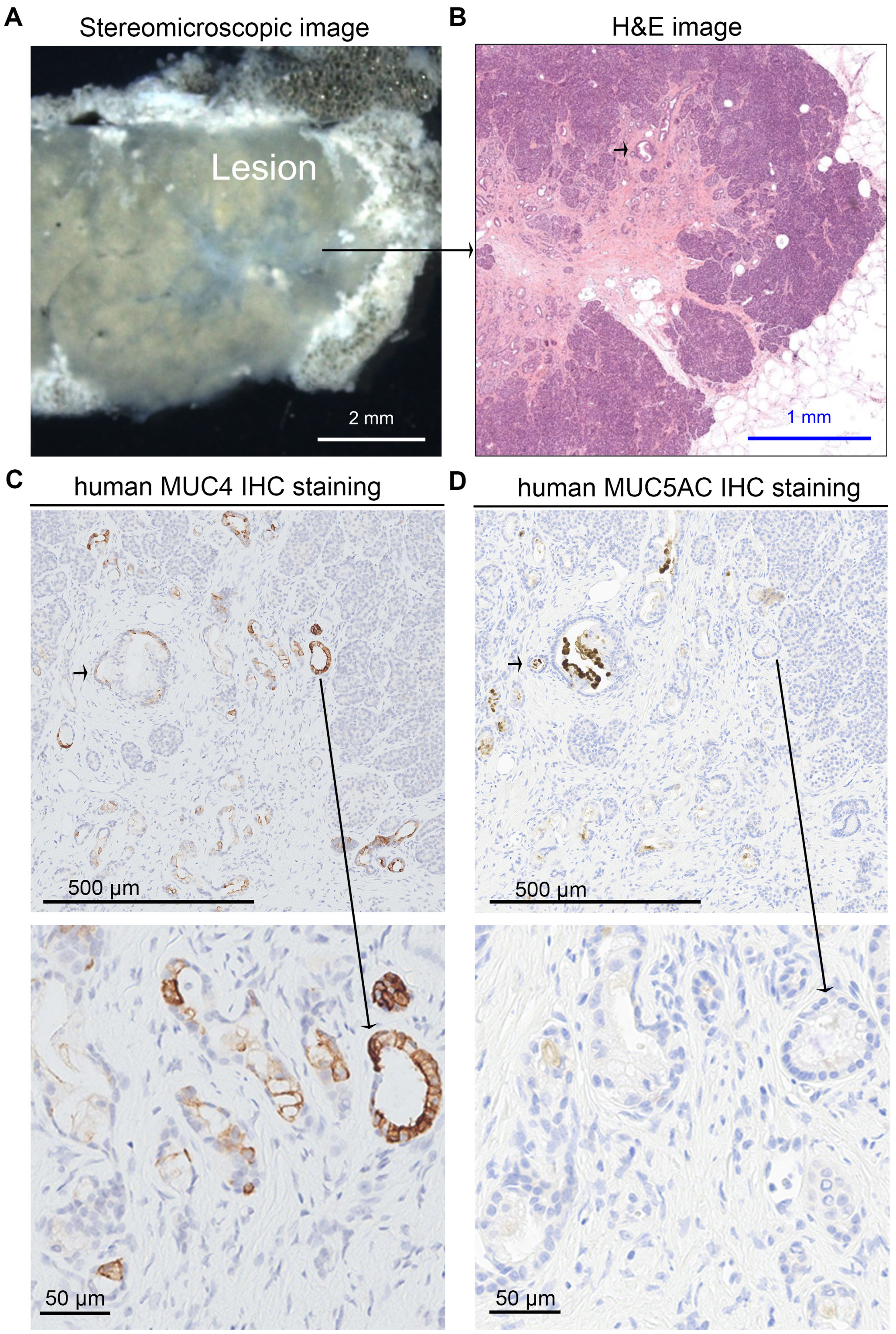

Figure S6. The majority of early human PanIN cells exhibit MUC4 expression while lacking MUC5AC. A) Stereomicroscopic image of the human pancreatic section with early PanINs. **B)** Confirmation of PanI by H&E staining. **C)** IHC staining of early human PanINs by anti-MUC4 antibody. Arrows indicate the same region with larger magnification. **D)** IHC staining of early human PanINs by anti-MUC5AC antibody. Arrows indicate the same region with larger magnification.

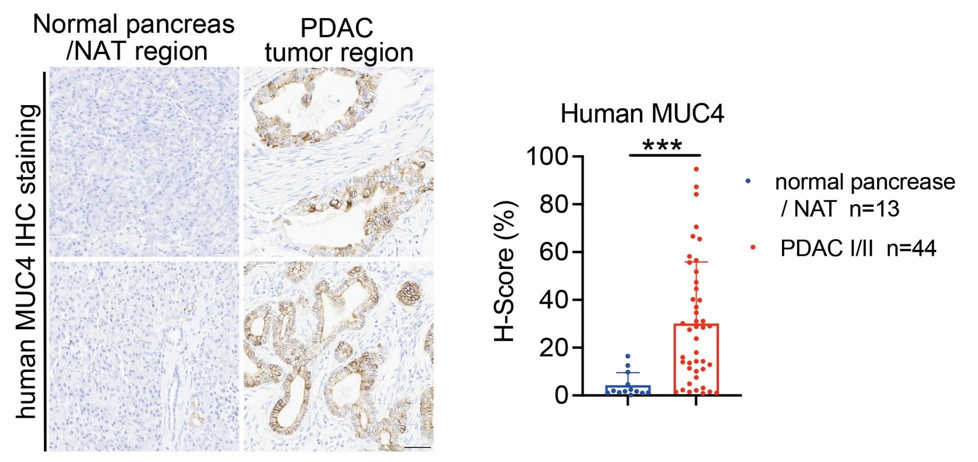

Figure S7. MUC4 overexpression in most PDAC specimens.

The IHC staining of human MUC4. Representative images (left panel) and quantification (right panel) of human MUC4 staining in normal pancreas tissue/ normal pancreas tissue adjacent to the tumor (NAT) and PDAC I/II samples from tissue arrays, PA483e and HPan060SC02 (BioMaxima S.A.). Bar, 50 µm. Each dot represents the datum from one person. Values were presented as mean ± SD. ***, $P < 0.001$ (two-tailed Student's t-test).

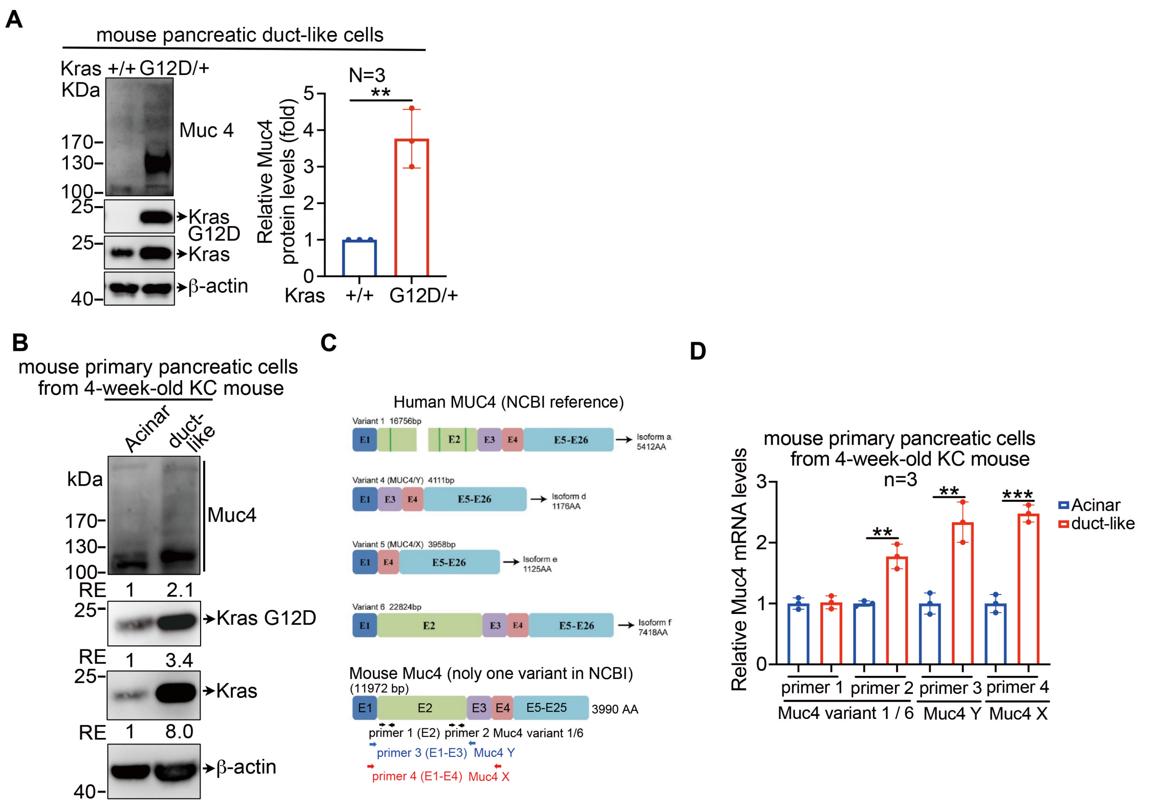

Figure S8. Short Muc4 variants are highly expressed in the pancreatic ductal-like cells with Kras$^{G12D/+}$.

A) Western blotting analysis of Muc4, Kras G12D, total Kras, and β-actin in the ductal-like cells of 4-week-old *Kras*$^{+/+}$ and *Kras*$^{G12D/+}$ mice (Left panel). Since MUC4 exhibits multiple variants, the shorter variants, MUC4/X and MUC4/Y, in humans have approximate molecular weights of 123.8 kDa and 129.4 kDa, respectively. Additionally, the presence of a band at 100 kDa is often attributed to non-specific binding. Hence, I quantified all bands above 100 kDa to determine the levels of mouse Muc4 expression. Relative Muc4 expression levels in *Kras*$^{G12D/+}$ cells are normalized to those from Kras$^{+/+}$ cells, and to the internal control, β-actin (Right panel). N indicates independent experiment. Values were presented as mean ± SD. **, $P < 0.01$ (two-tailed Student's t-test). **B)** Muc4 protein expression level in ductal-like cells was normalized to those from acinar cells, and to the internal control, β-actin. **C)** The NCBI database shows four human *MUC4* variants and one mouse *Muc4* variant. Based on human *MUC4* organization, four primer sets were designed for detecting mouse *Muc4* variants. **D)** The mRNA levels of *Muc4* variants in acinar to ductal-like cells were normalized to those in acinar cells, and to the amount of internal control gene, *GAPDH*. n indicates total measurements in the qPCR experiment. Each dot represents the datum from one measurement. Values were presented as mean ± SD. **, $P < 0.01$; ***, $P < 0.001$ (two-tailed Student's t-test).

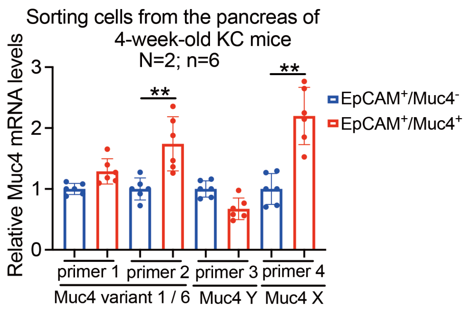

Figure S9. Double EpCAM/Muc4-positive pancreatic cells have Muc4/X overexpression. $EpCAM^+/Muc4^-$- or $EpCAM^+/Muc4^+$-pancreatic cells were isolated by FACS from 4-week-old KC mice and subjected to qPCR analysis of *Muc4* variants with primer sets as shown in fig. S5C. N indicates independent experiment, and n indicates total measurements in all experiments. Each dot represents the datum from one measurement. Values show mean ± SD. **, $P < 0.01$ (two-tailed Student's t-test).

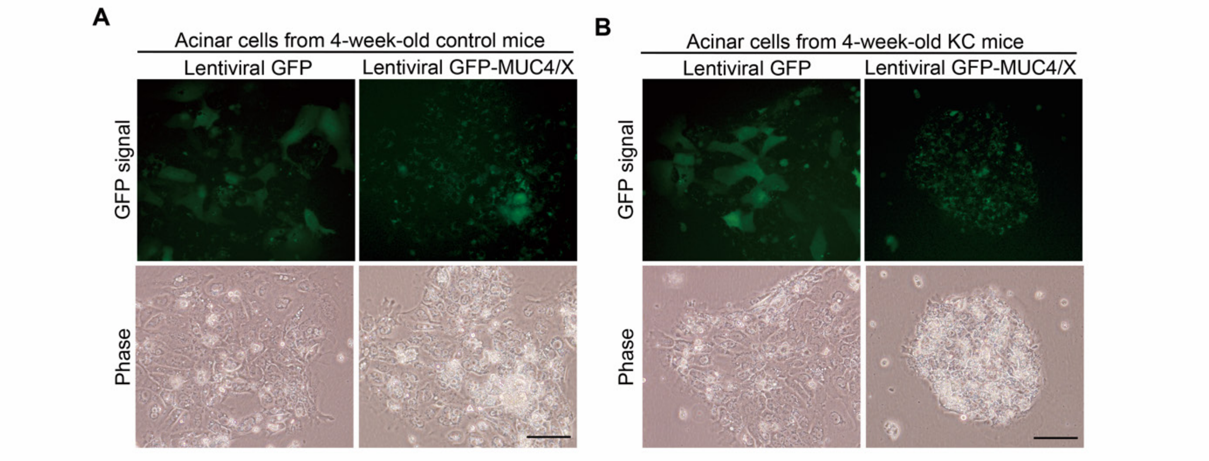

Figure S10. Ectopically expressing GFP or GFP-MUC4/X in primary pancreatic acinar cells.

A and B) Primary acinar cells were isolated from 4-week-old control **(A)** or KC **(B)** mice. After one day of culture, primary pancreatic acinar cells were infected with 10 MOI of lentiviral GFP or lentiviral GFP-MUC4/X overnight. After recovering from virus infection for one day, we enriched GFP-positive cells using 1 μg/mL puromycin selection for two days. Then, we allowed the cells to recover for one day before detecting GFP-positive cells using a fluorescent microscope. Bar, 100 μm.

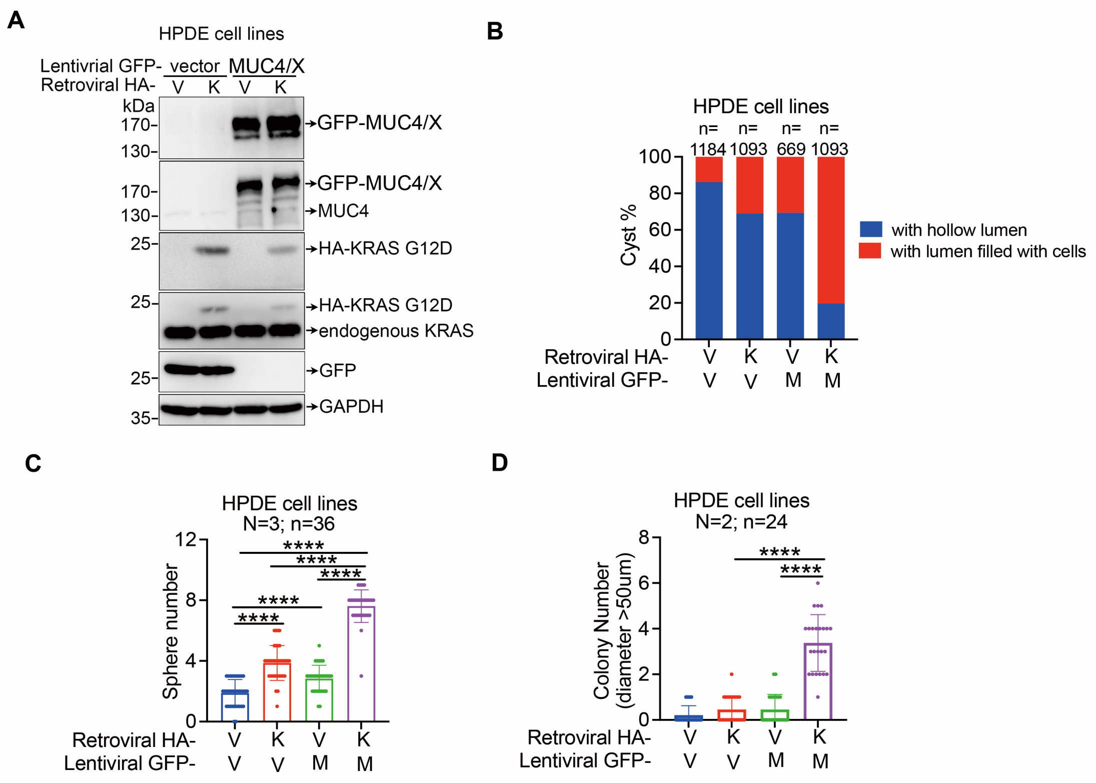

Figure S11. Co-expression of HA-KRASG12D and GFP-MUC4/X in HPDE cells promotes abnormal cyst formation and increases the number of spheroid formation and soft colony formation.

A-D) HPDE cells stably expressing indicated retroviral gene/ lentiviral gene were subjected to Western Blot analysis **(A)**, cyst formation analysis **(B)**, spheroid formation analysis **(C)**, and soft agar colony formation analysis (**D**). V, vector; K, $KRAS^{G12D}$; M, MUC4/X. N indicates independent experiments, and n indicates total cyst number or total measurements in all experiments. Each dot represents the datum from one measurement. Values were presented as mean ± SD. ****, *P*< 0.0001 (two-tailed Student's t-test).

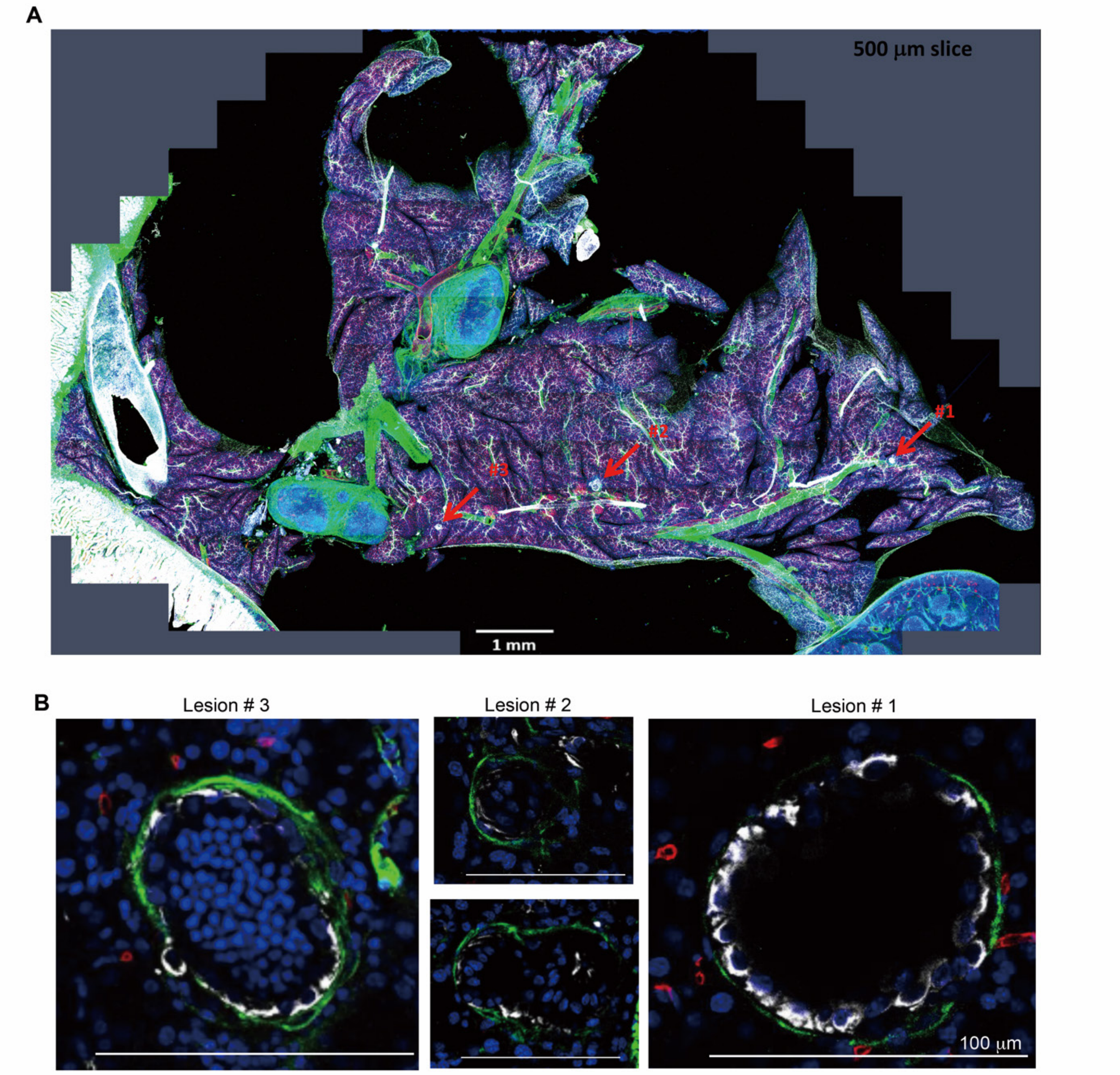

Figure S12. αSMA⁺ fibroblasts are associated with PanIN cells in the 4-week-old KC mouse.

A) The 3D imaging projection of a 500 µm pancreas slice. Bar, 1mm. Red arrows: PanINs. **B)** The enlarged lesion images. Blue signal: nucleus staining; White signal: CK19-staining (PanIN cells); Green signal: αSMA-staining (fibroblast); Red: germ agglutinin labeling (blood vessel). Bar, 100 µm

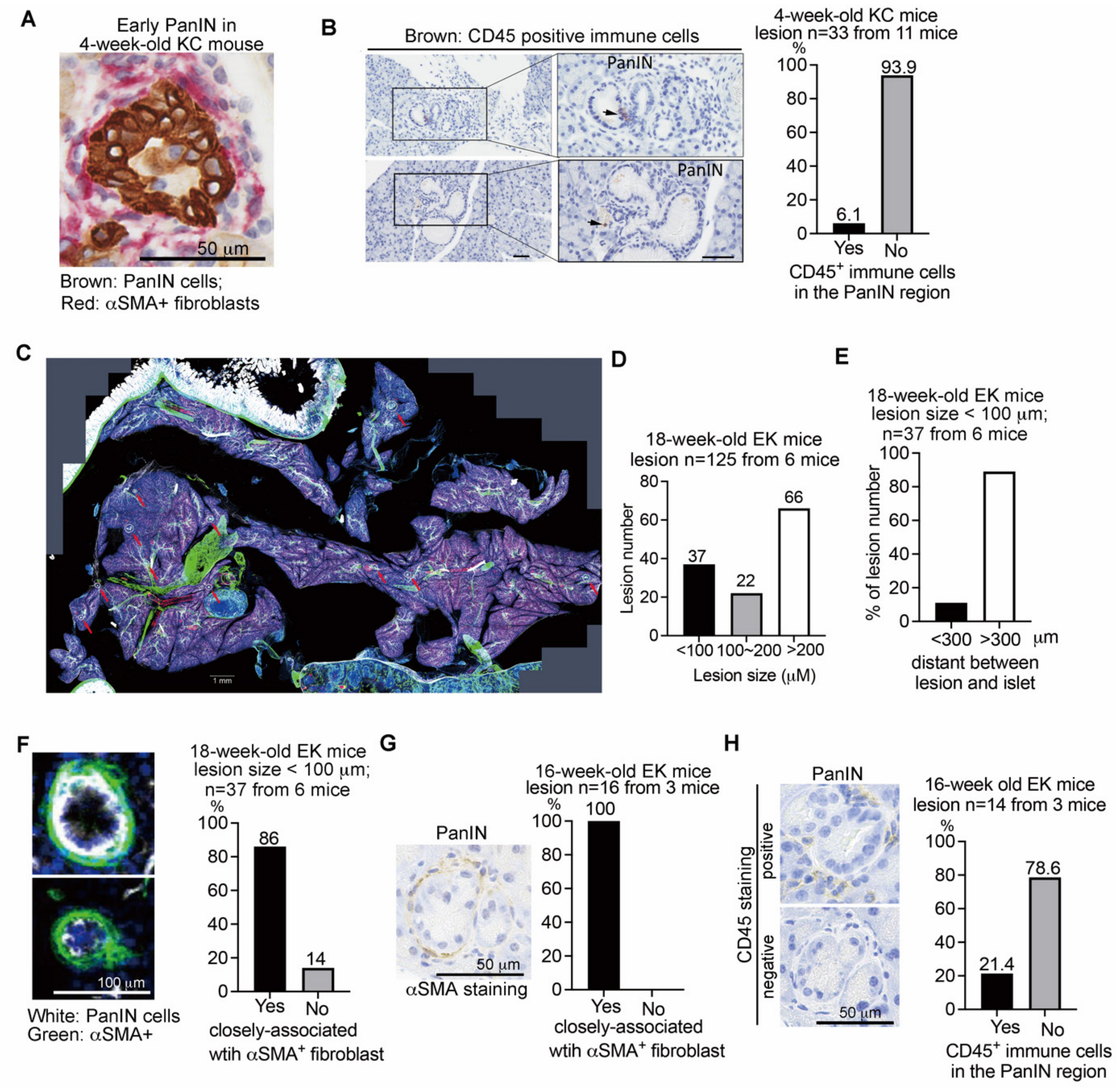

Figure S13. αSMA positive fibroblasts but not CD45$^+$ immune cells are closely associated with early PanIN cells.

A) Representative images of the IHC co-staining of early PanN cells with anti-CK19 antibody (brown color) and anti-αSMA antibody (red color) in 4-week-old KC mouse. Bar, 50 μm. **B)** Representative images of the IHC staining of immune cells with anti-CD45 antibody in 4-week-old KC mice (left panel) and quantification of CD45 staining (right panel). Bar, 50 μm. Each dot represents the datum from one mouse. Values show mean ± SD. **C)** The 3D imaging projection of a 500 μm pancreas slice. Bar, 1mm. Red arrows: PanINs. **D)** The size distribution of PanINs in 18-week-old EK mice. **E)** Quantification of the association of early PanINs (size< 100 μm) and islet in the pancreas of 18-week-old EK mice. An association is defined by the distance between lesion and islet within 300 μm. **F)** Representative images of 3D histology detected early PanINs in 18-week-old EK mice using a 500 μm section (left panel) and quantification of early PanINs associated with αSMA$^+$ fibroblasts (right panel). Blue signal: the nucleus staining; White signal: CK19-staining (PanIN cells); Green signal: αSMA$^+$ fibroblast

staining. Bar, 100 μm. **G)** Representative images of the IHC staining of fibroblasts with anti-αSMA antibody in 16-week-old EK mice (left panel) and quantification of αSMA^{+} fibroblasts associated with early PanIN cells (right panel). Bar, 50 μm. **H)** Representative images of the IHC staining of immune cells with anti-CD45 antibody in 16-week-old EK mice (left panel) and quantification of CD45 staining (right panel). Bar, 50 μm. Each dot represents the datum from one mouse. Values show mean ± SD.

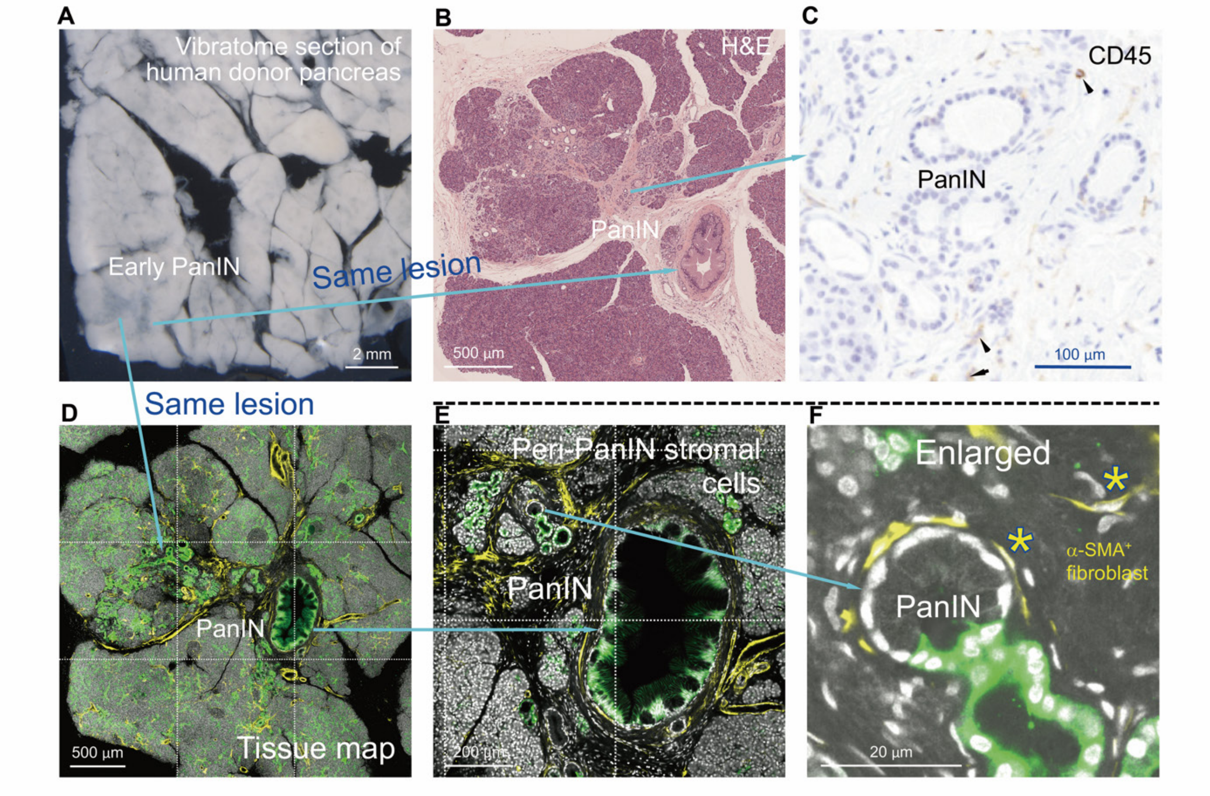

Figure S14. The early PanINs in human pancreatic tissue are closely-associated with α SMA+ fibroblasts but not CD45^{+} immune cells.

A) Stereomicroscopic image of the early lesion in human pancreas (sex/age (years)/BMI: male/50/20.3). Vibratome section, 350 µm in thickness. **B)** H&E image of the PanIN-1A lesion in the magnified region. **C)** The lesion is further enlarged and identified with CD45 (NCL-L-LCA, Leica Biosystems) staining of infiltrated leukocytes. Black arrows: immune cells. **D-F)** Paired α-SAM (yellow; MS-113-P1, Thermo) and CK7 (green; ab68459, Abcam) staining of peri-PanIN stromal accumulation. Tile-scanning and image stitching were used to generate the images. The α-SAM^{+} stromal cells are enlarged in *F* (asterisks). White signal: DAPI staining of nuclei. *A-F* examine the same microenvironment (arrows).

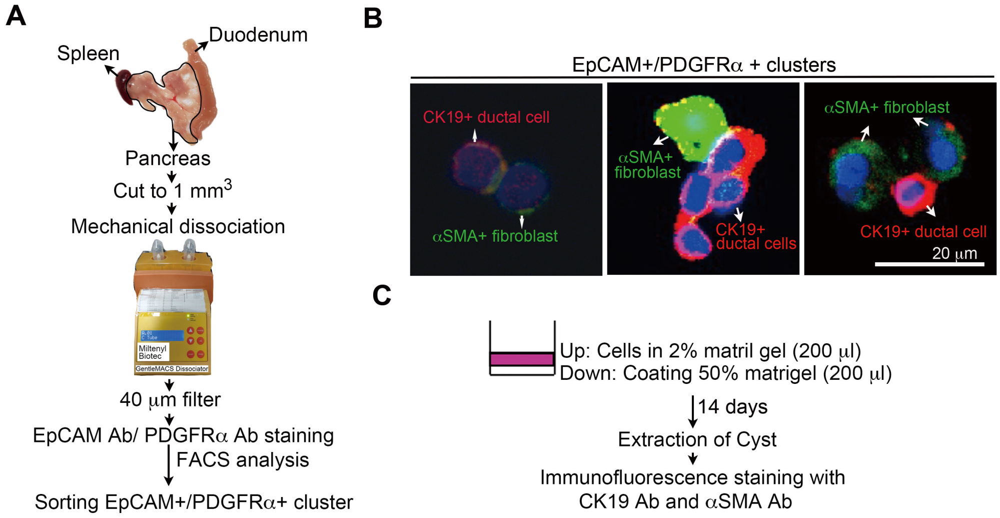

Figure S15. The procedures for cell cluster isolation and cyst staining.

A) The procedure for cell cluster isolation using MACS dissociation and FACS analysis with EpCAM/PDGFRα antibodies. **B)** The images of cell clusters after FACS analysis. Cells were from the pancreatic tissue of 4 weeks-old KC mouse. **C)** The procedures for cyst formation and cyst staining.

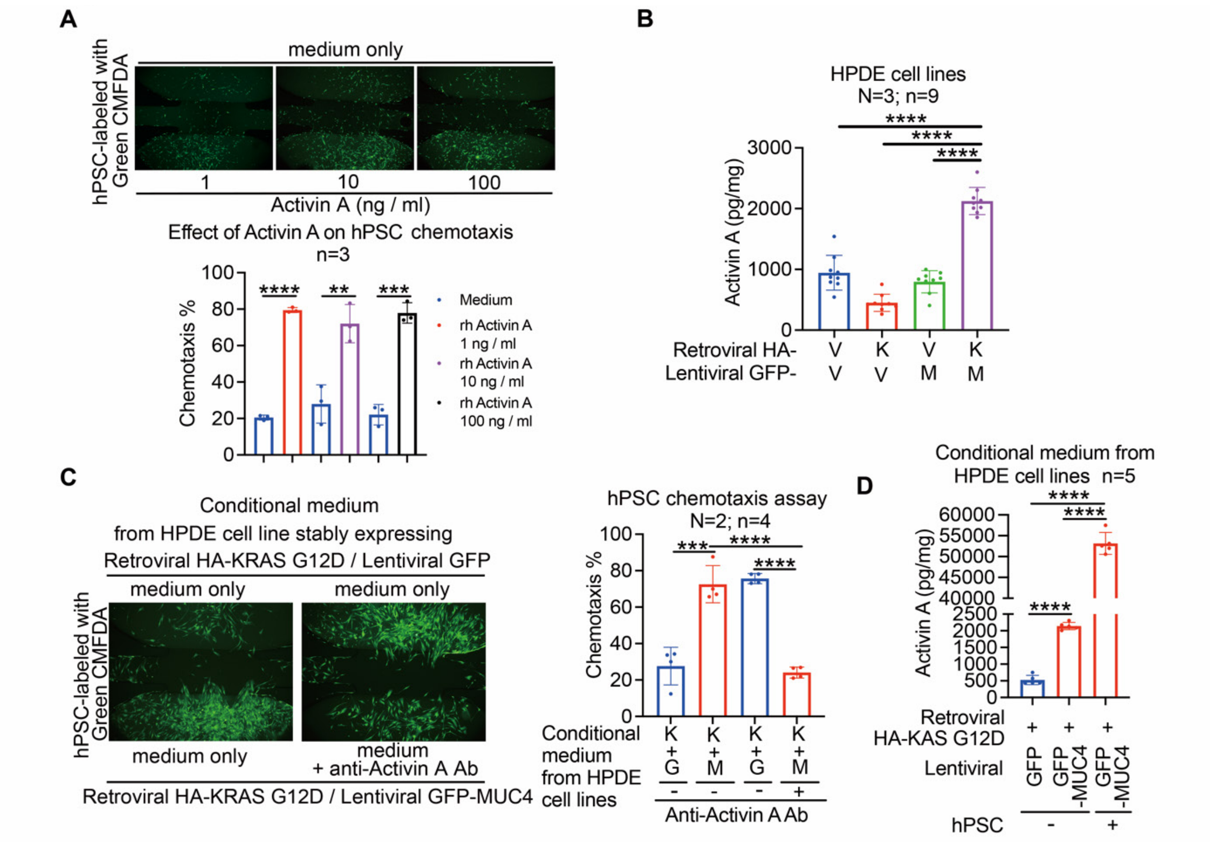

Figure S16. MUC4 overexpression promotes HPDE cells with Oncogenic KRAS^{G12D} to secrete Activin A for fibroblast recruitment.

hPSC were labeled with CellTracker^{TM} Green CMFDA for chemotaxis analysis. **A)** The effect of Activin A on hPSC chemotaxis was analyzed using µslide chemotaxis analysis. Representative images (upper panel) and quantification (bottom panel) of the chemotaxis effect. **B and C)** 4 x10^5 indicated HPDE cells were grown in the culture dishes for 48 hrs, and conditional media were harvested for the experiments. **(B)** Quantification of Activin A in the conditional medium using ELISA analysis. **(C)** The conditional media from indicated HPDE cells were pre-treated with or without 4 µg/ml of anti-Activin A antibody for 30 mins and subjected to µslide chemotaxis analysis. Representative images (left panel) of hPSC chemotaxis and its quantification (right panel). **D)** 4 x10^5 HPDE cells stably expressing indicated genes were co-cultured with 8 x10^5 fibroblasts for 48 hrs, and their conditional media were harvested for Activin A ELISA analysis. N indicates independent experiments, and n indicates total measurements in all experiments. Each dot represents the datum from one measurement. Values were presented as mean ± SD. **, P < 0.01; ***, P< 0.001; ****, P< 0.0001 (two-tailed Student's t-test).

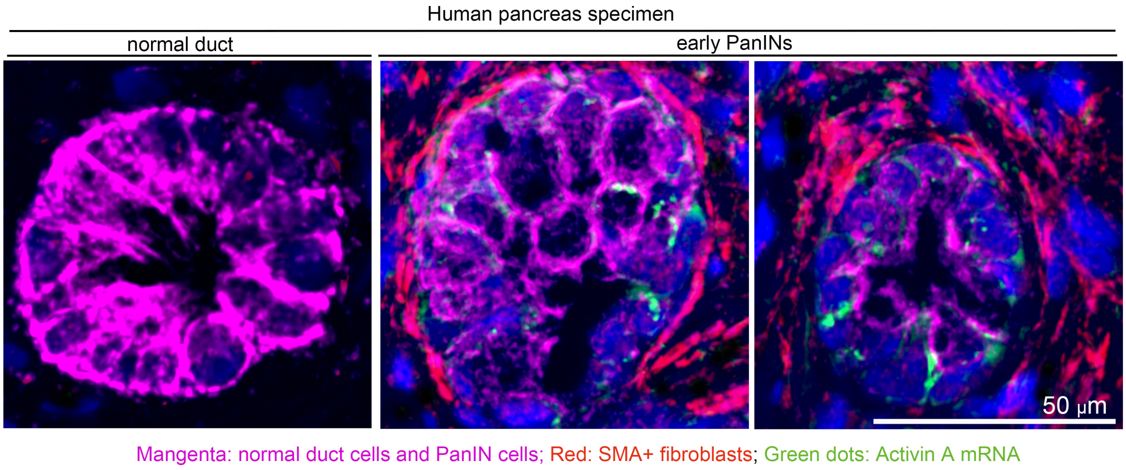

Figure S17. The Activin A mRNA was highly expressed in the early human PanIN cells. The human pancreatic section with normal ductal cells and early PanINs was used to perform Opal Multiplex IHC Assay. Mangeta: PanIN cells stained with anti-CK19 antibody; Red signal: αSMA^{+} fibroblasts stained with anti-αSMA antibody; Green dot signal: Activin A mRNA with dig-labeled antisense mRNA probes. Bar, 50 μm.

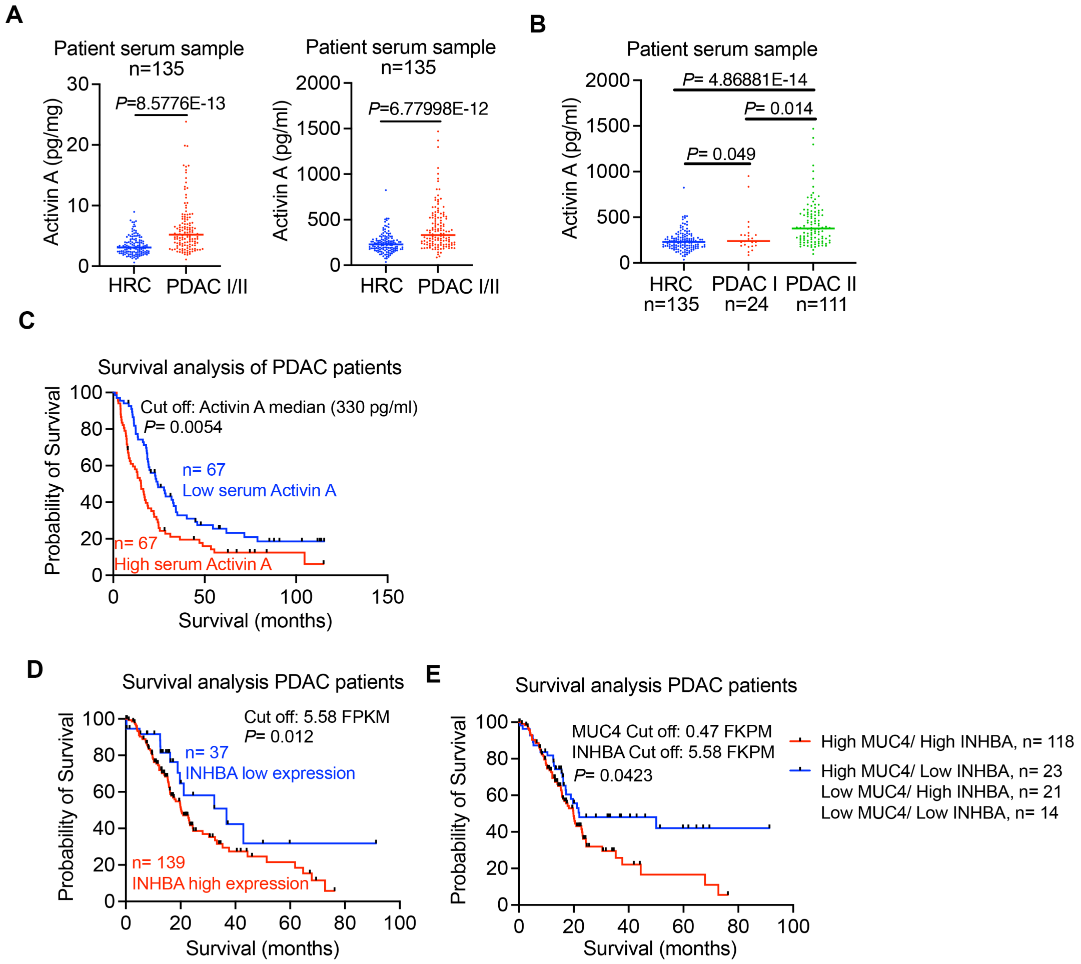

Figure S18. High serum Activin A levels are associated with PDAC stage and poor survival of PDAC patients.

The High-Risk Controls (HRCs) are individuals with a family history of PDAC who remained free from pancreatic malignancies throughout a follow-up period exceeding 2 years. **A and B)** Sera from HRC and stage I/II of PDAC patients were used to perform Activin A ELISA analysis. Each dot represents the datum from one person. Values were presented as mean ± SD. *P* value was calculated by two-tailed Student's t-test. **C)** Kaplan–Meier analysis of overall survival in patients with low or high serum Activin A levels. One patient with elevated serum Activin A levels was excluded from the analysis due to the absence of available survival data. **D and E)** Plots of the survival probability of pancreatic cancer patients with INHBA **(D)** and MUC4/ INHBA **(E)** mRNA expression levels from the Human Protein Atlas (MUC4: https://www.proteinatlas.org/ENSG00000145113-MUC4/pathology/pancreatic+cancer; INHBA: https://www.proteinatlas.org/ENSG00000122641-INHBA/pathology/pancreatic+cancer). *P* values in C to E are calculated by log-rank test.

Table S1. Cadaveric donor information

Sex	Age	BMI	Cause of death	Location of detected PanIN	Sample usage in this study
female	64	27.1	cerebrovascular/stroke	head of pancreas	Figure S5 and S16
male	50	20.3	brain tumor	tail of pancreas	Figure S13

Table S2. Quantitative real-time PCR primers

Mouse Muc4<br>Primer 1 (Located in exon 2)	Forward: 5' GACACCCATGTCCCTTTCCA 3'
	Reverse: 5' GGTTTGAGGTTTGCTGGTCT 3'
Mouse Muc4<br>Primer 2 (Located in exon 2)	Forward: 5' TCAATGCCCAGCACTTCTTCA 3'
	Reverse: 5' GAGGACGTTCCCCAAGACAT 3'
Mouse Muc4<br>Primer 3 (Located in exon 2-3)	Forward: 5' CACTGGAGAGTTCCCTGGCT 3'
	Reverse: 5' GGGCTAGTAAGGGTCGAGG 3'
Mouse Muc4<br>Primer 4 (Located in exon 2-4)	Forward: 5' CTCTGGAGCCATGAGAGGGC 3'
	Reverse: 5' AAAATCCACAGTCCTGGCAAAC 3'
Mouse Muc4<br>(Located in the β subunit)<br>for cell cluster experiment	Forward: 5' CCTCCTCTTGCTACCTGATGC 3'
	Reverse: 5' GGAACTTGGAGTATCCCTTGTTG 3'
Mouse Muc1	Forward: 5' GGCATTCGGGCTCCTTTCTT 3'
	Reverse: 5' TGGAGTGGTAGTCGATGCTAAG 3'
Mouse Muc5ac	Forward: 5' GTGGTTTGACACTGACTTCCC 3'
	Reverse: 5' CTCCTCTCGGTGACAGAGTCT 3'
Mouse Acta2 (αSMA gene)	Forward: 5' GGCACCACTGAACCCTAAGG 3'
	Reverse: 5' ACAATACCAGTTGTACGTCCAGA 3'
Mouse GAPDH	Forward: 5' CTTTGGCATTGTGGAAGGGC 3'
	Reverse: 5' CAGGGATGATGTTCTGGGCA 3'
Human ACTA2<br>(αSMA gene)	Forward: 5' GAGCGTGGCTATTCCTTCGT 3'
	Reverse: 5' TTCAAAGTCCAGAGCTACATAACACAGT 3'
Human GAPDH	Forward: 5' GGCTCTCCAGAACATCATCCCTGC 3'
	Reverse: 5' GGGTGTCGCTGTTGAAGTCAGAGG 3'

Table S3. Clinical demographic data in pancreatic ductal adenocarcinoma (PDAC) patients and high risk controls (HRC)

	**PDAC I-II (n = 135)**	**High risk Controls (n = 135)**
Age, mean (SD), years*	61.2 (14.4)	48.05 (14.05)
Sex (M/F)	75 (55.6%)/60 (44.4%)	53 (39.3%)/82 (60.7%)
TNM Stage		
I	24 (17.8%)	
II	111 (82.2%)	
Overall survival, median (SD), months	18.82 (28.24)	
TNM Stage I	44.93 (33.05)	
TNM Stage II †	16.87 (25.23)	

Data are presented as the mean (SD) or number. Survival data are presented as the median (SD).

*P < 0.0001 between pancreatic cancer patients and control group.

†P < 0.0001 between respectable pancreatic cancer patients and advanced pancreatic cancer patients.

PDAC, pancreatic ductal adenocarcinoma; HRC, High risk controls; M, Male; F, Female
